# Supplementary material for: Issues related to pregnancy, pregnancy prevention and abortion in the context of the COVID-19 pandemic: a WHO qualitative study protocol
Source: BMJ Open. 2022 Oct 6;12(10):e063317. doi: 10.1136/bmjopen-2022-063317 (PMC9539649; doi:10.1136/bmjopen-2022-063317)
Supplement: Supplementary data [file bmjopen-2022-063317supp001.pdf]

BMJ Publishing Group Limited (BMJ) disclaims all liability and responsibility arising from any reliance placed on this supplemental material which has been supplied by the author(s)

Legend of Supplementary files

Supplementary file S1: Study instrument--Interview Core Topic Guide

Supplementary file S2: Study instrument--Interview Detailed Topic Guide

Supplementary file S3: Written or oral informed consent form

Supplementary files:

Comment: Supplementary files 1 and 2 are only topic guides for helping interviewers to keep in mind the main content of the questions they should approach with each interviewee, and are not of course a questionnaire to be filled. Adaptations are possible and recommended for local and personal characteristics of the subject and language use can also be adapted for schooling level of the participant. Interview tools and the informed consent form would also need to be adjusted in settings where abortion is legally restrictive so that the questions are posed in a sensitive and non-judgemental manner. Given the inclusion of sensitive topics, it is essential that the study team implements all measures to protect the privacy and confidentiality of participants. The guidance document should provide potential options to countries on which questions to eliminate or modify to fit the local context. This should also be considered in the interview guide. Similar careful attention should be given to questions related to violence.

Cecatti JG, *et al. BMJ Open* 2022; 12:e063317. doi: 10.1136/bmjopen-2022-063317

Supplementary file S1: Study instrument--Interview Core Topic Guide

Qualitative research on women's reproductive and psychosocial health in the context of COVID-19 - Core Topic Guide

1. Demographic information
    - Age
    - Ethnicity
    - Marital status
    - Socio-economic status
    - Occupation
    - Education status
    - Religion
    - Place of residence and housing condition
    - Obstetric characteristics (no. of pregnancies, parity)

2. Knowledge about COVID-19
    - Cause (sexual transmission)
    - Signs and symptoms of COVID-19
    - Care-seeking for COVID-19 infection
    - Sources of information and trusted communication channels (v. rumors, misinformation) Experience of COVID-19 (direct / indirect)
    - Perception of risk about being infected by COVID-19

3. Knowledge about pregnancy-related issues during COVID-19 (for pregnant participants)
    - Perception of risk associated with COVID-19 infection during pregnancy
    - Care-seeking for COVID-19 infection during pregnancy
    - Management of pregnancy with suspected COVID-19 infection

4. Prevention of COVID-19
    - Ways to prevent being infected by COVID-19 (probe at individual, household, community levels)
    - Responsibility to prevent COVID-19 (probe at individual, household, community levels, authorities)
    - Religious / socio-cultural practices and influencers
    - Perceptions of the vaccine

5. Sexual and reproductive health
    - Normal practices (including use of condoms)
    - Decision-making and agency to act
    - Issues of access / utilization including antenatal care
    - Challenges faced
6. Contraceptive practices
    - Changes in practice (if any) due to COVID-19
    - Who / what influenced the changes (if no changes in practices, why not)
    - Is pregnancy being avoided / delayed due to COVID-19?

Cecatti JG, *et al. BMJ Open* 2022; 12:e063317. doi: 10.1136/bmjopen-2022-063317

- Access to/utilization of different methods of contraception (barriers / drivers)
- Challenges in access / utilization

7. Induced abortion
    - Perceptions about abortion at pre-COVID times
        - The role abortion plays in women's lives
        - Fear of stigma
        - Inequities in access to safe abortion
    - Perceptions about abortion during COVID-19
    - Influencing factors for perceptions and changes articulated
    - Perceptions of women if more women are seeking abortions due to COVID-19?
    - Methods of abortion (medical abortion or surgical abortion)
    - Access to different methods of abortion (barriers / drivers)
    - Challenges in accessing / utilizing safe abortion services (inequities in access)

8. Violence against women

For SRH healthcare providers
- Perceptions of changes in the nature and magnitude of violence against women, and especially domestic violence during COVID-19
- Nature of service provision during COVID-19, whether services for VAW are being changed or adapted and if safety considerations are adequately addressed?
- Access to /use of health services for VAW and related challenges

For pregnant and non-pregnant women
- Formal and informal systems of support, and coping strategies for VAW
- Perceptions of safety in home and in community safety/increased risk during COVID-19
- Women's expectations of services for gender-based violence
- What are additional stress factors (e.g. school-age children at home, older parents, loss of job/income, partner loss of job, etc.)
- How does stress affect frequency and severity of violence, particularly during COVID-19

9. Psycho-social
    - Psychosocial issues associated with being infected with COVID-19 (probe: more significant for women/men, more significant for pregnant women)

10. Final questions
    - On-going concerns about COVID-19 and its possible impact
    - Needs related to accurate information (probe further information needs – NB consider how to provide information if necessary)
    - Needs related to sexual reproductive health services (probe further SRH needs – NB consider how to support/provide if necessary)

Additional themes will be added as appropriate for each target group and questions revised accordingly, across the three phases of the study.

Cecatti JG, *et al. BMJ Open* 2022; 12:e063317. doi: 10.1136/bmjopen-2022-063317

Supplementary file S2: Study instrument--Interview Detailed Topic Guide

Qualitative research on women's reproductive and psychosocial health in the context of COVID-19

Interview detailed topic guide for pregnant women

1. Demographic information
- Age
- Sex
- Ethnicity
- Marital status
- Annual income
- Occupation
- Education status
- Religion
- Place of residence and housing condition
- How many pregnancies did you have including current pregnancy?

2. Knowledge about COVID-19
- What did you know about the mode of transmission of the COVID-19 virus?
- What did you know about the signs and symptoms of COVID-19?
- Did you know where and how to seek care for COVID-19 infection? If yes, where and how?
- What's your sources of information about COVID-19? With whom do you communicate about COVID-19? How did you distinguish between facts and rumours/misinformation? Have you ever trusted rumours and misinformation during COVID-19? Please specify. How did you figure out what you trusted was wrong?
- Were you affected by COVID-19? Was any of your family members or friends/colleagues affected by COVID-19? How did being affected/having closed ones affected by COVID-19 affect your pregnancy? If you or your closed ones weren't affected by COVID-19, how did the epidemic/pandemic affect your pregnancy in general?
- What's your perception of risk about being infected by COVID-19? How did you estimate the risk?

3. Knowledge about pregnancy-related issues during COVID-19
- What's your perception of risk associated with COVID-19 infection during pregnancy? Are you aware of any risks associated with getting COVID-19 as a pregnant women? Please specify.
- Did you know where and how to seek care for COVID-19 infection during pregnancy? If yes, where and how? If you were ever affected by COVID-19, tell me your care-seeking experience.
- What did you know about the management of pregnancy with suspected COVID-19 infection? If you were ever infected by COVID-19, how did the infection challenge the management of your pregnancy by any means?

4. Prevention of COVID-19

- What did you know about the ways to prevent being infected by COVID-19? What about at individual levels? What about at household levels? What about at community levels? How did you practice these ways of prevention?
- Whom in your family was mainly responsible to practice prevention as a household? How do you think you, your household, your community, and the authorities fulfilled the responsibility to prevent COVID-19?
- How did you practice the prevention of COVID-19 differently than what is suggested by professionals due to religious/socio-cultural reasons (e.g. having gatherings due to religious reasons).
- What do you know about the COVID-19 vaccine?

5. Sexual and reproductive health

- Who (you, your partner, or your family member) usually make decision for various sexual and reproductive health related issues? Who is usually the agency to act?
- Did you have access to SRH services needed during COVID-19? If yes, how did you access to them? If not, what was the barrier? What did you do?
- What SRH-related challenges did you face during COVID-19? How did you cope with the challenges?

6. Contraceptive practices

- What is your normal practice of contraception?
- Was there any change in contraceptive practice among you and your partner due to COVID-19?
- If yes, what's the change? Who / what influenced the changes? If no, why not?
- Is your current pregnancy being delayed due to COVID-19?
- Did you have access to contraceptive methods needed during COVID-19? How many kinds of contraceptive methods did you have access to? Was there any barrier? What drives you to use this/these particular contraceptive method(s)?
- What other challenges did you meet in accessing / utilizing contraceptive methods? How did you cope with the challenges?

7. Induced abortion

- What's your perceptions about abortion during COVID-19? If you need to have abortion during COVID-19, will you opt for it? What challenges do you foresee in having abortion during COVID-19?
- What's your perceptions about abortion at typical times or rather, prior to COVID-19
- What were the influencing factors for your perceptions and changes articulated?

    - We have seen the increased need for reproductive health services such as abortion during this pandemic. Have you or someone you know ever had an abortion before? What role did it play in your life?
    - Were you fear of stigma when you have/intend to have/think of abortion?
    - Did you experience any inequities when you needed to access safe abortion?

- Did you seek abortion services during COVID-19? If yes, how many types of abortion methods were available? Was there any barrier of seeking safe abortion services during COVID-19? What's the driver for you to have the abortion and to have this particular kind of abortion?

- What other challenges did you meet in accessing / utilizing safe abortion services? How did you cope with the challenges?

8. Psycho-social

- Did you experience stress/anxiety/depression during COVID-19? How would rate your psychosocial health during your pregnancy? What role did COVID-19 play in influencing your psychosocial health during your pregnancy? Was it more significant for you or your partner?

9. Violence against women

For pregnant and non-pregnant women

- Formal and informal systems of support, and coping strategies for VAW
- Perceptions of safety in home and in community safety/increased risk during COVID-19
- Women's expectations of services for gender-based violence
- What are additional stress factors (e.g. school-age children at home, older parents, loss of job/income, partner loss of job, etc.)
- How does stress affect frequency and severity of violence, particularly during COVID-19

10. Final questions

- Do you have any concerns about COVID-19 currently? What's the impact on you?
- Was your need for accurate information fulfilled during COVID-19? What kind of accurate information you needed was missing during COVID-19? What do you think is the best way for you to access accurate information during COVID-19?
- Do you think you needs for sexual and reproductive health services were fulfilled during COVID-19? What kind of SRH services you needed wasn't met during COVID-19? What do you think is the best way the society can support the delivery of SRH services during emergencies like COVID-19?

Interview topic guide for non-pregnant women

1. Demographic information

- Age
- Sex
- Ethnicity
- Marital status
- Annual income
- Occupation
- Education status
- Religion
- Place of residence and housing condition

2. Knowledge about COVID-19

- What did you know about the mode of transmission of the COVID-19 virus?
- What did you know about the signs and symptoms of COVID-19?
- Did you know where and how to seek care for COVID-19 infection? If yes, where and how?

- What's your sources of information about COVID-19? With whom do you communicate about COVID-19? How did you distinguish between facts and rumours/misinformation? Have you ever trusted rumours and misinformation during COVID-19? How did you figure out what you trusted was wrong?
- Were you affected by COVID-19? Was any of your family members or friends/colleagues affected by COVID-19?
- What's your perception of risk about being infected by COVID-19? How did you estimate the risk?

3. Prevention of COVID-19

- What did you know about the ways to prevent being infected by COVID-19? What about at individual levels? What about at household levels? What about at community levels? How did you practice these ways of prevention?
- Whom in your family was mainly responsible to practice prevention as a household? Do you think you, your household, your community, and the authorities fulfilled the responsibility to prevent COVID-19?
- How did you practice the prevention of COVID-19 differently than what is suggested by professionals due to religious/socio-cultural reasons (e.g. having gatherings due to religious reasons).
- What are your thoughts/perceptions of the vaccine?

4. Sexual and reproductive health

- Who (you, your partner, or your family member) usually make decision for various sexual and reproductive health related issues? Who is usually the agency to act?
- Did you have access to SRH services needed during COVID-19? If yes, how did you access to them? If not, what was the barrier? What did you do?
- What SRH-related challenges did you face during COVID-19? How did you cope with the challenges?

5. Contraceptive practices

- What is your normal practice of contraception?
- Was there any change in contraceptive practice among you and your partner due to COVID-19?
- If yes, what's the change? Who / what influenced the changes? If no, why not?
- Did you have access to contraceptive methods needed during COVID-19? How many kinds of contraceptive methods did you have access to? Was there any barrier? What drives you to use this/these particular contraceptive method(s)?
- What other challenges did you meet in accessing / utilizing contraceptive methods? How did you cope with the challenges?

6. Induced abortion (applying the same changes as above)

- What's your perceptions about abortion during COVID-19? If you need to have abortion during COVID-19, will you opt for it? What challenges do you foresee in having abortion during COVID-19?
- What's your perceptions about abortion at typical times or rather, prior to COVID-19
- What were the influencing factors for your perceptions and changes articulated?

  - We have seen the increased need for reproductive health services such as abortion during this pandemic. Have you or someone you know ever had an abortion before? What role did it play in your life?
  - Were you fear of stigma when you have/intend to have/think of abortion?
  - Did you experience any inequities when you needed to access safe abortion?
- Did you seek abortion services during COVID-19? If yes, how many types of abortion methods were available? Was there any barrier of seeking safe abortion services during COVID-19? What's the driver for you to have the abortion and to have this particular kind of abortion?
- What other challenges did you meet in accessing / utilizing safe abortion services? How did you cope with the challenges?

7. Psycho-social

- Did you experience stress/anxiety/depression during COVID-19? How would rate your psychosocial health? What role did COVID-19 play in influencing your psychosocial health? Was it more significant for you or your partner?

8. Violence against women

For pregnant and non-pregnant women

- Formal and informal systems of support, and coping strategies for VAW
- Perceptions of safety in home and in community safety/increased risk during COVID-19
- Women's expectations of services for gender-based violence
- What are additional stress factors (e.g. school-age children at home, older parents, loss of job/income, partner loss of job, etc.)
- How does stress affect frequency and severity of violence, particularly during COVID-19

9. Final questions

- What concerns do you have about COVID-19 currently? What's the impact on you?
- Was your need for accurate information fulfilled during COVID-19? What kind of accurate information you needed was missing during COVID-19? What do you think is the best way for you to access accurate information during COVID-19?
- Do you think your needs for sexual and reproductive health services were fulfilled during COVID-19? What kind of SRH services you needed wasn't met during COVID-19? What do you think is the best way the society can support the delivery of SRH services during emergencies like COVID-19?

Interview topic guide for partners of pregnant and non-pregnant women

1. Demographic information

- Age
- Sex
- Ethnicity
- Marital status
- Annual income
- Occupation
- Education status

- Religion
- Place of residence and housing condition
- How many pregnancies did your partner have including current pregnancy?

2. Knowledge about COVID-19

- What did you know about the mode of transmission of the COVID-19 virus?
- What did you know about the signs and symptoms of COVID-19?
- Did you know where and how to seek care for COVID-19 infection? If yes, where and how?
- What's your sources of information about COVID-19? With whom do you communicate about COVID-19? How did you distinguish between facts and rumours/misinformation? Have you ever trusted rumours and misinformation during COVID-19? How did you figure out what you trusted was wrong?
- Were you affected by COVID-19? Was any of your family members or friends/colleagues affected by COVID-19? How did being affected/having closed ones affected by COVID-19 affect your partner's pregnancy? If you or your closed ones weren't affected by COVID-19, how did the epidemic/pandemic affect your partner's pregnancy in general?
- What's your perception of risk about being infected by COVID-19? How did you estimate the risk?

3. Knowledge about pregnancy-related issues during COVID-19 (For participants whose partners were pregnant during COVID-19)

- What's your perception of risk associated with COVID-19 infection during pregnancy? Are you aware of any risks associated with getting COVID-19 as a pregnant women?
- Did you know where and how to seek care for COVID-19 infection during pregnancy? If yes, where and how? If your partner was ever affected by COVID-19, tell me your care-seeking experience.
- What did you know about the management of pregnancy with suspected COVID-19 infection? If your partner was ever infected by COVID-19, did the infection challenge the management of her pregnancy by any means?

4. Prevention of COVID-19

- What did you know about the ways to prevent being infected by COVID-19? What about at individual levels? What about at household levels? What about at community levels? How did you practice these ways of prevention?
- Whom in your family was mainly responsible to practice prevention as a household? Do you think you, your household, your community, and the authorities fulfilled the responsibility to prevent COVID-19?
- How did you practice the prevention of COVID-19 differently than what is suggested by professionals due to religious/socio-cultural reasons (e.g. having gatherings due to religious reasons).

5. Sexual and reproductive health

- Who (you, your partner, or your family member) usually make decision for various sexual and reproductive health related issues? Who is usually the agency to act?
- Did your partner have access to SRH services needed during COVID-19? If yes, how did she access to them? If not, what was the barrier? What did you and your partner do?
- What SRH-related challenges did you and your partner face during COVID-19? How did you cope with the challenges?

6. Contraceptive practices

- What is your normal practice of contraception?
- Was there any change in contraceptive practice among you and your partner due to COVID-19?
- If yes, what's the change? Who / what influenced the changes? If no, why not?
- Is your partner's current pregnancy being delayed due to COVID-19?
- Did you have access to contraceptive methods needed during COVID-19? How many kinds of contraceptive methods did you have access to? Was there any barrier? What drives you to use this/these particular contraceptive method(s)?
- What other challenges did you and your partner meet in accessing / utilizing contraceptive methods? How did you cope with the challenges?

7. Induced abortion

- What's your perceptions about abortion at normal times?
- Have your partner ever had abortion before? What role did it play in your life?
- What's your perceptions about abortion during COVID-19? If your partner need to have abortion during COVID-19, will you opt for it? What challenges do you foresee in having abortion during COVID-19?
- What were the influencing factors for your perceptions and changes articulated?
- Did your partner seek abortion services during COVID-19? If yes, how many types of abortion methods were available? Was there any barrier of seeking safe abortion services during COVID-19? What's the driver for you and your partner to have the abortion and to have this particular kind of abortion?
- What other challenges did you and your partner meet in accessing / utilizing safe abortion services? How did you cope with the challenges?

8. Psycho-social

- How did you and your partner experience stress/anxiety/depression during COVID-19?
- If your partner is pregnant, how would you rate your and your partner's psychosocial health during her pregnancy? What role did COVID-19 play in influencing your and your partner's psychosocial health during her pregnancy? Was it more significant for you or your partner?
- If your partner is not pregnant, how would rate your and your partner's psychosocial health? What role did COVID-19 play in influencing your and your partner's psychosocial health? Was it more significant for you or your partner?

9. Final questions

- What concerns about COVID-19 do you have currently? What's the impact on you?
- Was your need for accurate information fulfilled during COVID-19? What kind of accurate information you needed was missing during COVID-19? What do you think is the best way for you to access accurate information during COVID-19?
- Do you think your and your partner's needs for sexual and reproductive health services were fulfilled during COVID-19? What kind of SRH services you needed wasn't met during COVID-19? What do you think is the best way the society can support the delivery of SRH services during emergencies like COVID-19?

Interview topic guide for health care professionals

1. Demographic information
- Age
- Sex
- Ethnicity
- Marital status
- Annual income
- Occupation
- Education status
- Religion
- Place of residence and housing condition

2. Knowledge about COVID-19
- What did you know about the mode of transmission of the COVID-19 virus?
- What did you know about the signs and symptoms of COVID-19?
- Did you know where and how to seek care for COVID-19 infection? If yes, where and how?
- What's your sources of information about COVID-19? With whom do you communicate about COVID-19? How did you distinguish between facts and rumours/misinformation? Have you ever trusted rumours and misinformation during COVID-19? How did you figure out what you trusted was wrong?
- Were you affected by COVID-19? Was any of your family members or friends/colleagues affected by COVID-19?
- What's your perception of risk about being infected by COVID-19? How did you estimate the risk?

3. Knowledge about pregnancy-related issues during COVID-19
- What's your perception of risk associated with COVID-19 infection during pregnancy?
- Where and how can people seek care for COVID-19 infection during pregnancy?
- What did you know about the management of pregnancy with suspected COVID-19 infection?

4. Prevention of COVID-19
- What did you know about the ways to prevent being infected by COVID-19? What about at individual levels? What about at household levels? What about at community levels?

5. Sexual and reproductive health
- Where and how can people seek SRH services needed during COVID-19? How was the utilization of SRH services in your hospital / clinic?
- What challenges did you meet in providing SRH services during COVID-19?

6. Contraceptive practices
- Where and how can people seek contraceptive methods during COVID-19? How many kinds of contraceptive methods were available in your hospital / clinic during COVID-19? How was the utilization of contraceptive services in your hospital / clinic?
- What challenges did you meet in providing contraceptive methods during COVID-19?

7. Induced abortion

- What's your perceptions about abortion at normal times?
- What's your perceptions about abortion during COVID-19?
- What were the influencing factors for your perceptions and changes articulated?
- Are more women seeking abortions due to COVID-19?
- How many methods of abortion are available in your hospital / clinic during COVID-19 and now? What kind of abortion do you suggest during COVID-19 and now? Why?
- What challenges did you meet in providing safe abortion services during COVID-19?

8. Psycho-social

- How did you experience stress/anxiety/depression during COVID-19? How would rate your psychosocial health? What role did COVID-19 play in influencing your psychosocial health?

9. Violence against women

For SRH healthcare providers

- Perceptions of changes in the nature and magnitude of violence against women, and especially domestic violence during COVID-19
- Nature of service provision during COVID-19, whether services for VAW are being changed or adapted and if safety considerations are adequately addressed?
- Access to /use of health services for VAW and related challenges

10. Final questions

- What concerns about COVID-19 do you have currently? What's the impact on you?
- What accurate information do you think should be provided to the society during COVID-19? What do you think is the best way to provide accurate information during emergencies like COVID-19?
- What sexual and reproductive health services do you think should be provided during COVID-19? What do you think is the best way the society can support the delivery of SRH services during emergencies like COVID-19?

For centres deciding on the inclusion of GBV in the main topics to be assessed in the study:

I'd like to hear your thoughts on how violence against women, and especially violence in the home, may be changing during this pandemic and under movement restrictions, and how the health system is responding to violence during the crisis.

A. Have you noticed any change in the number of women coming into the health centre reporting gender-based violence since the COVID-19 crisis began?
   - Follow up: Increase or decrease?
   - Follow up: Have the women coming in who report experiencing violence mostly been returning patients, or mostly been new patients?
   - Follow up: Have you noticed an increase in the frequency of abuse that women are experiencing since movement restrictions were put in place?
   - If possible, specify types of violence (physical, sexual, psychological by a partner), non-partner sexual abuse, other

B. Do you think that the violence women have been experiencing has become more severe

during the crisis, for example has there been an increase in injuries? Violence during pregnancy?

- Follow up: Have the types of violence that women are reporting changed? How so?

C. Since the start of COVID-19, has your health centre changed or adapted services for survivors ‘of violence?

- Probe: (if not mentioned) In some health centres, providers have maintained ‘case management’ using video calls, or text messages. Did your health centre make use of any new technologies to maintain services? If so did you develop a protocol to ensure safety of survivors?

D. Some health centres have had to deal with shortages of personal protective equipment and medications that have impacted their ability to offer services during the COVID-19 crisis. Have there been any new barriers to delivering services to survivors at your health centre?

E. In a lot of places, external referral systems have also been impacted by the crisis, such as lower numbers of available spaces in shelters due to the need for physical distancing, or NGOs that have been temporarily shut down. To your knowledge, have any changes have been made to your external referral systems? What are these?

- Follow up: Are any of the services to which you typically refer survivors available in a more limited capacity, or no longer available due to the crisis?

Cecatti JG, *et al. BMJ Open* 2022; 12:e063317. doi: 10.1136/bmjopen-2022-063317

Supplementary file S3: Written or oral informed consent form
Informed consent (the original version will be presented in local language)

Model 1 (pregnant women)

Part One: Information Sheet

We would like to invite you to participate in the study "Qualitative Research on Pregnancy, Pregnancy Prevention and Induced Abortion in the Context of COVID-19". Doctor X is the investigator responsible for the study. The project focuses in analysing the psychosocial impacts of COVID-19 epidemic in reproductive decisions and health care for women, partners and health professionals. The researchers are particularly interested in comprehend the needs, attitudes and practices related to family planning / contraception, abortion, views and experience with violence, and health care during and after pregnancy in the context of the COVID-19 epidemic.

If you decide to participate in the study you will be asked to be interviewed in a series of two interviews - one will be carried out at recruitment and the second one at six months later. If you prefer for practical or security reasons to have the interview performed by telephone, this option will be followed. The interview is planned to be tape recorded. The interviewers will ask about the short- and long-term impacts of COVID-19 on your experience of pregnancy, pregnancy prevention, and induced abortion etc. The interview will be conducted at a convenient time and place for you. Each interview will last approximately 60-90 minutes. You will receive all the information needed before, during and after the completion of the research, and we assure you that your name will not be disclosed, or any information that can identify you or your family. The study and the corresponding data will not allow identification of the women interviewed. In this sense, during the process of writing down the content recorded during the interviews all identifying data will be omitted to guarantee that you cannot be identified. The interviews will also be numbered in a way that will not allow identification of individual participants. The recordings of the interviews will be put in a safe way and sent to an online location to be stored after the interview. Transcriptions will be kept under an online service for storing data, accessible only to the research team. All tape recordings will be kept in a secure place. We will remove the information that would allow your identification, and because of that, the record of the interviews will not be destroyed. These data will be kept for a period according to the local national rules. By giving your consent for this study, you are also allowing us to use the data you share with us to inform other ongoing and future initiatives in the field.

Your participation is voluntary. Eventually a small amount of money will be given to you to cover your expenses to attend the interview. You are free to refuse to participate, and you may withdraw your consent or discontinue participation at any time. If for any reason you discontinue your participation, your data collected until this moment can be used, otherwise, if you withdraw your consent, your data will be excluded and will not be used. The refusal to participate will not result in any penalty or loss of benefits. You can skip any questions you do not want to answer. All of the research data will be under the care of the principal investigator.

Possible risks: We are asking you to share with us some very personal and confidential information, and you may feel uncomfortable talking about some of the topics. You do not have to answer any question or take part in the discussion/interview/survey if you don't wish to do so, and that is also fine. You do not have to give us any reason for not responding to any question, or for refusing to take part in the interview.

Study benefits: At the end of the first round of the project, the research team will disseminate through online platform with information on family planning, sexual and reproductive health. The study will not directly benefit the participant, and any resources made available will also be available if they do not consent to participate.

This project has been reviewed and approved by the (*name of the local IRB*). If you have questions right now, we will be happy to clarify. During the research you can also contact (*name of one research assistant*) at telephone number *(cell phone number available for questions)* or at email address *(e-mail address available for questions)* for any questions.

Part Two: Certificate of Consent

I have read the foregoing information, or it has been read to me. I have had the opportunity to ask questions about it and any questions I have been asked have been answered to my satisfaction. I consent voluntarily to be a participant in this study

Print Name of Participant__________________
Signature of Participant ___________________
Date ____________________________
Day/month/year

If illiterate

I have witnessed the accurate reading of the consent form to the potential participant, and the individual has had the opportunity to ask questions. I confirm that the individual has given consent freely.

Print name of witness____________ Thumb print of participant
Signature of witness_____________
Date _________________________
Day/month/year

Statement by the researcher/person taking consent

I have accurately read out the information sheet to the potential participant, and to the best of my ability made sure that the participant understands that the following will be done:

1. Semi-structured interview

I confirm that the participant was given an opportunity to ask questions about the study, and all the questions asked by the participant have been answered correctly and to the best of my ability. I confirm that the individual has not been coerced into giving consent, and the consent has been given freely and voluntarily.

A copy of this ICF has been provided to the participant.
Print Name of Researcher/person taking the consent________________________
Signature of Researcher /person taking the consent__________________________
Date ____________________________
Day/month/year

Cecatti JG, *et al. BMJ Open* 2022; 12:e063317. doi: 10.1136/bmjopen-2022-063317

Model 2 (non-pregnant women, partners and health care professionals)

Part One: Information Sheet

We would like to invite you to participate in the study "Qualitative Research on Pregnancy, Pregnancy Prevention and Induced Abortion in the Context of COVID-19". Doctor X is the investigator responsible for the study. The project focuses in analysing the psychosocial impacts of COVID-19 epidemic in reproductive decisions and health care for women, partners and health professionals. The researchers are particularly interested in comprehend the needs, attitudes and practices related to family planning / contraception, abortion, views and experience with violence and health care in the context of the COVID-19 epidemic.

If you decide to participate in the study you will be asked to be interviewed. The interview will be conducted at a convenient time and place for you. The interview will be held in one meeting, and will last approximately 60-90 minutes. If you prefer for practical or security reasons to have the interview performed by telephone, this option will be followed. The interview is planned to be tape recorded. You will receive all the information needed before, during and after the completion of the research, and we assure you that your name will not be disclosed, or any information that can identify you or your family. The study and the corresponding data will not allow identification of the women interviewed. In this sense, during the process of writing down the content recorded during the interviews all identifying data will be omitted to guarantee that you cannot be identified.. The interviews will also be numbered in a way that will not allow identification of individual participants. The recordings of the interviews will be put in a safe way and sent to an online location to be stored after the interview. Transcriptions will be kept under an online service for storing data, accessible only to the research team. All tape recordings will be kept in a secure place. We will remove the information that would allow your identification, and because of that, the record of the interviews will not be destroyed. These data will be kept for a period according to the local national rules. By giving your consent for this study, you are also allowing us to use the data you share with us to inform other ongoing and future initiatives in the field.

Your participation is voluntary. Eventually a small amount of money will be given reimbursement you to cover your incidental expenses to attend the interview. You are free to refuse to participate, and you may withdraw your consent or discontinue participation at any time. If for any reason you discontinue your participation, your data collected until this moment can be used, otherwise, if you withdraw your consent, your data will be excluded and will not be used. The refusal to participate will not result in any penalty or loss of benefits. You can skip any questions you do not want to answer. All of the research data will be under the care of the principal investigator.

Possible risks: We are asking you to share with us some very personal and confidential information, and you may feel uncomfortable talking about some of the topics. You do not have to answer any question or take part in the discussion/interview/survey if you don't wish to do so, and that is also fine. You do not have to give us any reason for not responding to any question, or for refusing to take part in the interview.

Study benefits: At the end of the first round of the project the research team will disseminate through online platform with information on family planning, sexual and reproductive health. The study will not directly benefit the participant, and any resources made available will also be available if they do not consent to participate.

This project has been reviewed and approved by the (*name of the local IRB*). If you have questions right now, we will be happy to clarify. During the research you can also contact (*name of one research assistant*) at telephone number *(cell phone number available for questions)* or at email address *(e-mail address available for questions)* for any questions.

Part Two: Certificate of Consent

I have read the foregoing information, or it has been read to me. I have had the opportunity to ask questions about it and any questions I have been asked have been answered to my satisfaction. I consent voluntarily to be a participant in this study

Print Name of Participant__________________
Signature of Participant ___________________
Date ____________________________
Day/month/year

If illiterate

I have witnessed the accurate reading of the consent form to the potential participant, and the individual has had the opportunity to ask questions. I confirm that the individual has given consent freely.

Print name of witness____________ Thumb print of participant
Signature of witness_____________
Date _________________________
Day/month/year

Statement by the researcher/person taking consent

I have accurately read out the information sheet to the potential participant, and to the best of my ability made sure that the participant understands that the following will be done:

1. Semi-structured interview

I confirm that the participant was given an opportunity to ask questions about the study, and all the questions asked by the participant have been answered correctly and to the best of my ability. I confirm that the individual has not been coerced into giving consent, and the consent has been given freely and voluntarily.

A copy of this ICF has been provided to the participant.
Print Name of Researcher/person taking the consent________________________
Signature of Researcher /person taking the consent__________________________
Date ____________________________
Day/month/year

Cecatti JG, *et al. BMJ Open* 2022; 12:e063317. doi: 10.1136/bmjopen-2022-063317
