# Supplementary material for: Issues related to pregnancy, pregnancy prevention and abortion in the context of the COVID-19 pandemic: a WHO qualitative study protocol
Source: BMJ Open. 2022 Oct 6;12(10):e063317. doi: 10.1136/bmjopen-2022-063317 (PMC9539649; doi:10.1136/bmjopen-2022-063317)
Supplement: Author's manuscript [file bmjopen-2022-063317.draft_revisions.pdf]

BMJ Open is committed to open peer review. As part of this commitment we make the peer review history of every article we publish publicly available.

When an article is published we post the peer reviewers' comments and the authors' responses online. We also post the versions of the paper that were used during peer review. These are the versions that the peer review comments apply to.

The versions of the paper that follow are the versions that were submitted during the peer review process. They are not the versions of record or the final published versions. They should not be cited or distributed as the published version of this manuscript.

BMJ Open is an open access journal and the full, final, typeset and author-corrected version of record of the manuscript is available on our site with no access controls, subscription charges or pay-per-view fees (http://bmjopen.bmj.com).

If you have any questions on BMJ Open's open peer review process please email info.bmjopen@bmj.com

BMJ Open

Issues related to Pregnancy, Pregnancy Prevention and Abortion in the Context of the COVID-19 pandemic: a WHO qualitative study protocol

Journal:	*BMJ Open*
Manuscript ID	bmjopen-2022-063317
Article Type:	Protocol
Date Submitted by the Author:	26-Mar-2022
Complete List of Authors:	Cecatti, Jose; University of Campinas, Obstetrics and Gynecology<br>Bahamondes, Luis; State University of Campinas – UNICAMP, Obst & Gynecol<br>Ali, Moazzam; Organisation mondiale de la Sante,<br>Alangea, Deda Ogum; University of Ghana College of Health Sciences, Population, Family & Reproductive Health<br>Brizuela, Vanessa; WHO, Department of Reproductive Health and Research<br>Nahyuha Chomi, Eunice; Institut de Recherche en Sciences de la Sante<br>Kouanda, Seni; Institut de Recherche en Sciences de la Sante<br>Karmaliani, Rozina; Aga Khan University School of Nursing & Midwifery, AKUSONAM<br>Ladak, Laila; Aga Khan University, School of Nursing and Midwifery<br>Lumbiganon, Pisake; Khon Kaen University, Faculty of Medicine<br>Emefa, Modey; University of Ghana<br>Jen, Sothornwit; Khon Kaen University, Department of Obstetrics and Gynaecology<br>Kuganantham, Hamsadvani; World Health Organization, Department of Reproductive Health and Research<br>Kim, Caron; World Health Organization, Sexual and Reproductive Health and Research<br>Collaborator Group, WHO HRP Social Science Research Team; World Health Organization
Keywords:	Reproductive medicine < GYNAECOLOGY, COVID-19, QUALITATIVE RESEARCH

SCHOLARONE™
Manuscripts

RESEARCH PROTOCOL

Issues related to Pregnancy, Pregnancy Prevention and Abortion in the Context of the COVID-19 pandemic: a WHO qualitative study protocol

Jose Guilherme Cecatti [1]
Luis Bahamondes [1]
Moazzam Ali[2]
Deda Ogum Alangea[3]
Vanessa Brizuela[2]
Eunice Chomi[4]
Seni Kouanda[4]
Rozina Karmaliani[5]
Laila Ladak[5]
Pisake Lumbiganon[6]
Emefa J Modey[3]
Jen Sothornwit[6]
Hamsa Kuganantham[2]
Caron R Kim[2]

* On behalf of WHO HRP Social Science Research Team

1. Department of Obstetrics and Gynaecology from the University of Campinas and Cemicamp, Center for Research in Reproductive Health of Campinas, Brazil.
2. *UNDP-UNFPA-UNICEF-WHO-World Bank Special Programme of Research, Development and Research Training in Human Reproduction (HRP), a cosponsored programme executed by the World Health Organization (WHO)*, Geneva, Switzerland.
3. Dept. of Population, Family & Reproductive Health, School of Public Health, College of Health Sciences, University of Ghana, Akkra, Ghana.
4. Institut de Recherche en Sciences de la Sante, Ouagadougou, Burkina Faso.
5. Aga Khan University School of Nursing and Midwifery, Karachi, Pakistan.
6. Department of Ob & Gyn, Faculty of Medicine, Khon Kaen University, Khon Kaen, Thailand.

Corresponding author*:

Caron R Kim (kimca@who.int)
UNDP/UNFPA/UNICEF/WHO/World Bank Special Programme of Research, Development and Research Training in Human Reproduction (HRP)
Department of Sexual and Reproductive Health and Research
World Health Organization
Avenue Appia 20
Geneva, Switzerland

For peer review only - http://bmjopen.bmj.com/site/about/guidelines.xhtml

ABSTRACT

Introduction: The World Health Organisation (WHO) has generated standardised clinical and epidemiological research protocols to address key public health questions for SARS-CoV-2 (COVID-19) pandemic. We present a standardised protocol with the aim to fill a gap in understanding the needs, attitudes and practices related to sexual and reproductive health (SRH) in the context of COVID -19 pandemic, focusing onrelated to pregnancy, pregnancy prevention and abortion.

Methods and analysis plan: This protocol is a prospective qualitative research, using semi-structured interviews with at least 15 pregnant women at different gestational ages and after delivery, 6 months apart from the first interview. At least 10 male partners, 10 non-pregnant women and 5 healthcare professionals will be interviewed once during the course of the research. Higher number of subjects may be needed if a saturation is not achieved with these numbers. Data collection will be performed in a standardised way by skilled trained interviewers using written notes or audio-record of the interview. The data will be explored using the thematic content analysis and the researchers will look for broad patterns, generalisations, or theories from these categories.

Ethics and dissemination: The current protocol was first technically assessed and approved by the WHO scientific committee and then approved by its Ethics Review Committee as a guidance document. It is expected that each country/setting implementing such a generic protocol adapted to their conditions also obtain local ethical approval.

Comments for the user's consideration are provided in grey boxes throughout the document, as the user may need to modify methods slightly because of the local context in which this study will be carried out.

Key words: sexual and reproductive health; COVID-19 pandemic; psychosocial health; qualitative study

For peer review only

Strengths and limitations of this study:

- This is part of a series of WHO generic protocols with the purpose of facilitating and standardising the assessment of SRH issues during and after the COVID-19 pandemic in low and middle-income countries.
- With a generic protocol, adaptations of it are essential and encouraged, depending on the characteristics and resources from the countries/settings where they are planned to be implemented or other places deciding to do the same.
- One study limitation would be the difficulties and risks of different contexts of participating centres´ places regarding the COVID-19 pandemic at the moment of study implementation, which could introduce biases into subjects' perception.

For peer review only

INTRODUCTION

According to the World Health Organization (WHO), as of 26 September 2021, around 232 million cases and 4.7 million deaths of SARS-CoV-2 (Covid-19) cases have been confirmed globally. COVID-19 was declared as a public health emergency of international concern (PHEIC) at the end of January and characterized as a pandemic by WHO on 11st March 2020 [1].

China was the first country heavily affected by the pandemic since December 2019 [2], soon followed by several others in Asia, and then successively in Europe, America and Africa. After the efforts in preventing and controlling the pandemic have been consolidated and expanded, with some positive signs of recovery from the pandemic in several countries, currently a second wave (and in some cases a third wave) has initiated everywhere, before a vaccine could be readily available for the population [1]. To restrain the spread of COVID-19, domestic mobility was strictly regulated and in some affected areas transportation was restricted, with some differences in strategies adopted by different countries in different timeframes. In addition, physical distancing measures and isolation were firmly recommended in many countries and territories in different degrees, including closure of some basic services such as in-person classes at schools and universities, business activities, commerce, industry and even some healthcare services. As a result, there are some information indicating a disrupted healthcare service provision, including services in sexual and reproductive health (SRH) [3,4]. Despite the enormous medical and research resources directed towards the COVID-19 response, there are several gaps in the knowledge with regards to how the virus affects the body, even after recovery, and how the disease and the pandemic has affected people's lives.

Given SARS-CoV-2 novelty as virus of human transmission, there is limited evidence available to identify the impact of COVID -19 on SRH. At the start of the pandemic, there were questions whether or not the transmission of the virus or disease occurs from the pregnant woman to the fetus including at the early stage of fetal development [5]. The evidence has been growing on the risk of mother-to-child transmission, whether during pregnancy, childbirth or breastfeeding. [6-9]. In any case, the potential effects of COVID-19 infection during pregnancy on maternal and fetal health would influence women, their partners and healthcare professionals' decisions on reproductive healthcare.

For peer review only

For peer review only - http://bmjopen.bmj.com/site/about/guidelines.xhtml

According to the UNFPA, as health systems became overwhelmed with COVID-19 cases, clinical staff may not have had the time or personal protective equipment needed to provide contraceptive guidance and provision [10]. Women were and are refraining from visiting healthcare facilities due to movement restrictions or fears about COVID-19 exposure. Supply chain disruptions are limiting the availability of contraceptives in many places. It is anticipated that significant levels of lockdown-related disruption over the months since the pandemic was declared could leave more than 47 million women in low- and middle-income countries unable to use modern contraceptives, leading to a projected 7 million additional unintended pregnancies [10].

In places in which medical termination of pregnancy is allowed, it has also been suggested that the demands for these services, including information provision have increased, which may be related to lack of contraceptive commodities or fear of unknown consequences of infection during pregnancy in the context of pandemic. There is some evidence from lower-resourced settings of women, especially married or in union women, having less control over the decision-making process of contraception or abortion [11,12]. Their husbands/partners and the extended family members have more control over their reproductive autonomy. It is important to examine how COVID-19 and the lack of resources during the pandemic influenced the decision-making process and the power dynamics involved in accessing contraception and abortion using information already available or implementing surveys for this [13,14].

Thus, it is important to assess the behavioural and psychosocial impacts of the COVID-19 pandemic on some aspects of women's SRH, especially among those who are pregnant, who are planning to become pregnant and those who want to avoid pregnancy, together with their partners and healthcare professionals, with a special attention to domestic and gender-based violence [15].

The current community-based protocol covers various SRH issues: abortion, contraception, pregnancy, sexual transmitted diseases (STD) prevention, domestic and gender-based violence (GBV), stigma, and discrimination. Covering multiple aspects of SRH will provide a better understanding of the *status quo* of SRH under the COVID-19 pandemic.

Comment: This section should be updated with country/setting-specific and updated data on COVID-19 epidemiology and current research findings prior to submission to local/national

For peer review only

Institutional Review Boards (IRB) or donor agencies. Specific information regarding the health system management and provision of services should also be included in this section (facility-based childbirths, access to safe and legal abortion services, and access to contraception).

Problem statement, aims and objectives*:*

The global research question is on what is the impact of COVID-19 on the SRH of women and their partners. The main aim is to increase knowledge and understanding of the behavioural and psychosocial impacts of the pandemic on women and men's SRHR, especially those related to pregnancy, pregnancy prevention and abortion.

Their specific objectives are: 1) to investigate how the existence of the COVID-19 pandemic affects women's knowledge, attitudes, needs and practices on SRH services such as pregnancy care, pregnancy prevention, and abortion; 2) to explore how the pandemic affects men's and healthcare professionals' attitudes, needs and practices related to pregnancy, pregnancy prevention, and abortion; 3) to understand the knowledge and the perceptions about COVID-19 impact for the study population and how this relates to their SRH-related attitudes and behaviours.

Comment: The SRH topics that the local study team intends to explore should be inserted in the objectives and may vary widely depending on the characteristics of the setting. Additional secondary objectives can be included, depending on the local context, in addition to the willingness of researchers and availability or resources to expand the current proposal.

METHODS

Study design

This study will use a phenomenological qualitative design with semi-structured interviews to collect data in either one point in time (for the male or female partner and health workers) or two points in time (for women), 6-months apart. It is planned to be conducted in selected urban, semi-urban and possibly rural regions and will recruit a purposive sample of pregnant and non-pregnant women to enable researchers to explore women's knowledge, attitudes and practices in different temporal and situational contexts under COVID-19, including diversity in pregnancy status, SRH needs, and socioeconomic status [16]. In addition, their partners (males or females) and local healthcare professionals will also be recruited to participate.

For peer review only - http://bmjopen.bmj.com/site/about/guidelines.xhtml

Study settings

Comment: there should be a brief description of the location – country, state, province, city – where the study is planned to be implemented, with some arguments justifying why this choice was made. Inform where and how pregnant women have access to antenatal care, where and how they deliver and access postnatal care, and if, where and how they get access to abortion and family planning services. State the number of health facilities chosen and their locations.

This protocol was developed to be implemented mainly in low and middle-income countries with institutions that have traditionally been partners in research with the WHO/HRP. However, any country or research centre can use the same protocol and they will be very welcomed. To capture variation in responses, health facilities in both rural and/or semi-urban/urban settings will be selected as focal points, including primary health unit or hospital where pregnant women are usually required to seek antenatal services. This enables the researchers' easy access to individuals attending the facility for interviews. The focal facilities will be selected purposively, using such criteria as available resources, COVID-19 pandemic status, availability of SRH services and the willingness of the healthcare providers to participate. The final selection of study sites will be confirmed jointly by the research team and local authorities, depending on the national procedures and regulations.

Comment: The implementation protocol should clearly define the catchment area of included health facilities and describe the demographics of the study sample. The strategy for getting help from health care providers at health facilities or other ways of identifying eligible subjects will rely on local characteristic of the health system and should be detailed described.

Study participants and sampling

The healthcare providers of the facilities selected as focal points will be asked to help the research team identify potential participants attending the facility. Pregnant women will be recruited at the local healthcare clinics when they access antenatal and/or SRH care services. Other participants, i.e., non-pregnant women, partners of pregnant and non-pregnant women and healthcare professionals, will be recruited from these same selected facilities during clinical visits. To explore contraceptive choices, abortion care and pregnancy care, different women will need to be selected accordingly. Therefore, the location sampling method (venue

For peer review only - http://bmjopen.bmj.com/site/about/guidelines.xhtml

based) will be used to recruit the participants [16], where participants can be selected from places they attend, which can be identified in advance. This location sampling process is easily implemented in healthcare facilities where a sufficient number of ~~required~~ available participants for the study are supposed to be present at an acceptable period of time.

Researchers will approach these women and men following standard inclusion criteria. For pregnant women, the inclusion criteria include (1) age 18-45 years old; (2) living within the catchment area of the healthcare facility; (3) who were pregnant (ideally, in early pregnancy, in the first trimester) during the COVID-19 outbreak in that specific location (Level 3-4 on the WHO Situational Level Assessment); (4) either who have tested positive or negative for SARS-CoV-2 but currently not with an active infection (i.e., current PCR+ cases or within 2 weeks from the beginning of symptoms will not be recruited in the study while in this condition). For non-pregnant women, the inclusion criteria include: (1) aged 18-45 years old; (2) living within the catchment area of the facility; (3) who are not pregnant during and immediately after the COVID-19 outbreak in that location at the moment of enrolment; and (4) who are attending the health facility with the purpose of getting any service on SRH~~.~~ Women who do not meet the above criteria or who do not wish to take part will be excluded. Meanwhile, partners/family members of women in both groups attending the healthcare facility as companions and healthcare professionals from these facilities will be invited to participate in the interviews. Participation will be voluntary and only individuals who consent to participate after receiving information will be enrolled. Informed consent will be administered to the study participants in their local language highlighting the details about the study objectives and processes. Signed informed consents forms will be mandatory for face-to-face interviews. Otherwise, if interview is performed by phone or other electronic means, a verbal consent will be obtained; this will be necessary if the participant is not literate or has visual impairment. To account for the possible loss to follow-up of pregnant women, we will recruit 20% more participants than the intended sample size during the first interview.

Comment: The study will use gatekeepers who will be health service providers not participating in the study or community members who are known and trusted by the clients. Their main role will be to limited to identification and introduction of potential participants to the study team.

Sample size calculation

This study will recruit a sample of a minimum of 15 pregnant women per facility. Specifically, the suggested sample size is an approximation to the lower bound (the minimum number or

For peer review only - http://bmjopen.bmj.com/site/about/guidelines.xhtml

participants required). However, the upper bound is determined by sampling until saturation, which means they should be selected until it is found that similar answers are given to similar questions with no new themes emerging. The decision on saturation is expected to be taken according the experienced and trained interviewers/research assistants under the supervision of the local PI and other possible skilled participating researchers. Sample sizes can vary largely depending on the context that could interfere with the saturation of gathered information. In addition, at least 10 non-pregnant women, 10 partners (any gender, for both pregnant and non-pregnant women), and 5 healthcare professionals will also be recruited in each country. The minimum sample sizes were not determined by any specific criterion, but using a priori estimation. This was empirically decided as the minimum number of subjects in each category to provide meaningful information to be gathered with that from other countries/settings. A purposive sampling strategy aimed at saturation on the central study themes is appropriate for a qualitative study.

Comment: the sample size estimation should roughly follow the above-mentioned process, but adaptations are possible depending on the local characteristics and timeframe relative to the COVID-19 pandemic.

Adjustments should also be made if testing for infection is unavailable. Recruitment can be undertaken with assessment of infection status based on history (ie lack of symptoms or no report of being in close contact with someone with COVID-19)

Patient and public involvement

There was no patient or public involvement in the design of this protocol.

Data collection methods

This study includes a prospective series of two semi-structured interviews to be conducted with pregnant women and one semi-structured interview to be conducted with non-pregnant women, partners of both pregnant and non-pregnant women, and healthcare professionals in selected study areas. The reason for conducting two interviews with pregnant women is to explore the short-term and long-term impact and changes of antenatal care, abortion and potential experiences with childbirth. Each interview will last approximately 60 – 90 minutes. The script of the interviews will follow a topic guide and the researchers will promote a safe, convenient and comfortable environment to enable a comprehensive rapport. The topic-guide will be pre-tested to ensure question validity and assess interview length. During the interview, the research team will ask open-ended questions to participants and record field notes.

The process of participant recruitment aims to provide comprehensive information about the study as well as to build trust and rapport with the participants, as an essential step for

For peer review only - http://bmjopen.bmj.com/site/about/guidelines.xhtml

them to make a fully informed and voluntary decision. This process will be documented with a signed written or oral recorded informed consent. The contact information of the enrolled pregnant women will be asked (e.g., phone number or other forms of preferred contact), as well as that of other contacts (e.g., relatives) during the first interview to improve chances for follow-up for the second interview. Follow-up and the second interview will be performed in person or through phone/teleconference based on the participant's preference and need.

The interviews will ask about fertility desires, pregnancy, pregnancy prevention, abortion, and their related needs for accurate information and reproductive services. The first interview will capture how the above-mentioned factors, especially the needs for reproductive services were affected by COVID-19. The second interview will assess how the above-mentioned factors changed during the outbreak. The semi-structured interview allows for exploration of other SRH issues if these come up. The interviews will document pregnant women's knowledge, concerns, needs, attitudes and practices throughout the duration of their pregnancy on SRH, abortion, contraception, gender-based violence (GBV), etc.

A single semi-structured interview will be conducted with the other participants at one point in time. Interviews with non-pregnant women will document their knowledge, attitudes and practices towards SRH under the context of COVID-19. Interviews with partners will explore their knowledge, attitudes and concerns for their own and partner's SRH and wellbeing, including abortion. Depending on the context, local researchers may wish to interview extended family members (such as in-laws) in addition or instead of partners, as indicated by local customs. Interviews with healthcare professionals will focus on access to and availability of contraception and safe abortion; health-systems versus women's roles and responsibilities for prevention of pregnancy in the context of COVID-19; health system capacity to provide high-quality care for women; attitudes about pregnancy, contraception and abortion in the context of COVID-19; and perceived psychosocial effects on women, their families and local communities.

The study instruments were adapted from a core master guide built by WHO for qualitative research on women's reproductive and psychosocial health. In particular, questions were adapted to the context of COVID-19, but they should also include items relevant to the local settings. The instrument will be translated into the language of the location and pre-tested to ensure its validity and reliability with adjustments if needed. The interview core and detailed guides are present in Supplementary file S1 and S2.

For peer review only - http://bmjopen.bmj.com/site/about/guidelines.xhtml

Interviewers may have to confront difficult situations amongst infected participant cohort – like death or serious complications from COVID-19. The pandemic has changed the way the people live, exposed most of the population to unsafe and difficult situations which has affected many people personally (either because they themselves were sick or their close ones, or because they might have lost close ones to the disease). Similar sensitive situations could also appear regarding GBV and child abuse for instance. Ideally the study team members should have access to a mental healthcare provider for counselling and support to researchers and interviewees experiencing difficult situations. The country coordinating team should be able to provide the participants in the study with appropriate recommendations on how and where to access specific services if problems arise during the interviews.

Several changes occurred in the dynamics of the pandemic since its beginning. The protocol will probably be implemented in several different countries experiencing different stages of the pandemic. This is a potential factor to enrich the content of the findings from different settings. In addition, current experience on clinical and research activities show that such an interview is possible with personal protective equipment (PPE) or using interviewers who have already had COVID-19 or who were already vaccinated.

Comment: Depending on the local experience and availability of resources, the interviews should ideally be recorded and then appropriate data management with records, transcription, use of specific software and related topics should be adapted in the current protocol. If audio-recording is not an option, then data collection and recording of the interview will need to be adapted using hand notes. Additional themes can be added to the interview as appropriate for each target group and questions revised accordingly, across the whole study. Interview tools and the informed consent form would also need to be adjusted in settings where abortion is legally restrictive so that the questions are posed in a sensitive and non-judgemental manner. Given the inclusion of sensitive topics, it is essential that the study team implements all measures to protect the privacy and confidentiality of participants. The guidance document (supplementary files) should provide potential options to countries on which questions to eliminate or modify to fit the local context. This should also be considered in the interview guide. Similar careful attention should be given to questions related to violence.

Data Analyses

For peer review only - http://bmjopen.bmj.com/site/about/guidelines.xhtml

We adopt concepts from the Ecological Theory [17] to approach this research question. The Ecological Theory suggests that individual characteristics as well as the environment act interactively on one's personal development. In this context, individual characteristics refer to one's social-demographic characteristics, and one's knowledge and attitude related to SRH. The environment refers to a broad range of social and structural factors, including societal value, and access to SRH services. COVID-19 is the overarching environmental factor of the proposed study. This model serves as the framework for studying the knowledge, needs, attitudes and practices related to contraception, abortion, and healthcare before, during and after pregnancy in the context of the COVID-19 pandemic. This model will also guide study design, data collection, analysis and interpretation.

An inductive analytical technique will be used to analyse the data collected. Detailed examination of documents from interviews, either from written forms or transcription from recordings, will be conducted in order to generate initial categories and, above all, to suggest relationships between categories. Two researchers will do an initial screening of the interview transcripts and develop codes. During the process, the researchers will compile a list of themes and write short notes about important issues and patterns that are emerging. From these new themes, the researchers establish inductive codes. The researchers will then go through the transcripts again, and re-code them according to the new codes and themes. The researchers will also compile all the above-mentioned codes into a codebook. Two researchers will analyse the data concurrently either manually or with the help of a qualitative analysis software (such as NVivo®). The coding method and progress will be systematically discussed between the researchers and a third team member to ensure inter-coder reliability (ICR).

There are several optional techniques available for identifying core categories and their integration, such as data usage diagrams and written memos. Figure 1 provides a diagram with the elements of the procedures and process of applying the conceptual framework to this qualitative study.

There are different parties involved in this study as individuals living in COVID-19 pandemic areas during pregnancy, the SRH needs of non-pregnant women and their partners, including safe sex, contraception and abortion, as well as SRH healthcare providers. The participating partners (whether a man or a woman) in the study will be asked about their own SRH needs as well as those of their pregnant/not-pregnant partner.

For peer review only - http://bmjopen.bmj.com/site/about/guidelines.xhtml

Although the analysis is planned to provide a general full picture of SRH for women and men across all the participating countries/sites, the conceptual analysis will not be standardised across diverse contexts/countries due to their specificities and probable local adaptations for implementing the research protocol.

Data management and data access

Audio recordings and/or notes from the interviews will be transcribed verbatim, cross-checked as soon as feasible following the interview. Only the research team will have access to the data during the study. Single interviews will be de-identified at the time of transcription. Prospective interviews will be de-identified, transcribed, cross-checked, and summarised in order to inform subsequent interviews in the two-interview series. A participant ID will be assigned to each participant to ensure that data from the same participant is linked across two interview series. Data and transcriptions will be kept in encrypted hard drives, accessible only to the research team. All audio recordings will be kept in a secure place for the period of time as required for local country regulations and will then be destroyed. A subsample of the interview transcriptions will be translated into English (for those performed in a different language) to enable monitoring and inputs from WHO staff and consultants. All identifiable information, including telephone number and electronic accounts, which are used for contacting subjects during the interview, will be stored in a separate encrypted hard drive.

Ethics and dissemination

Respondents will provide a signed informed consent, also allowing eventually for a telephone interview (Supplementary file S3). The information collected will be de-identified. The identifiable information will be stored in a separate encrypted hard drive. The data will be kept in a safe storage way for the time as required by local national rules and laws on this topic. If additional analyses are planned, an appropriate permission should be obtained from the local ethics committees. If a participant decides to withdraw, she/he will be discontinued and data already collected will be used. However, if she/he withdrawn the consent, the respective data will be excluded and will not be used in the analysis.

Although the study is not planning to interview subjects currently with an active PCR+, all safety measures should be taken to minimise transmission risks. Masks will always be a requirement for both researchers and study participants and possibly other PPE and physical

For peer review only

distancing as locally required. In cases of suspected infection in either the researcher or the interviewee, they should be immediately referred to the health facility to be screened and tested, and the interview should be interrupted or postponed. If GBV is being assessed and women are identified in such a condition, social and legal advice should also be provided following the recommendations of local IRBs. In sites where abortions are illegal, discussions of access to abortion or post-abortion care may include risks to participants in terms of potential personal or legal repercussions.

There will be no financial incentives provided to participate in this study. However, the participants and their companions could receive compensation to cover the incidental costs of their participation, depending on the local regulations and availability of resources. This study protocol has received scientific approval through HRP's research project review panel (RP2) and ethics review committee (ERC) and, in accordance to local regulations, should be reviewed by a local ethics committee.

The results will be used to build an understanding of knowledge, practices, needs and attitudes related to fertility desires, pregnancy, pregnancy prevention and abortion under the context of COVID-19. They can help to improve health policies and SRH services. We aim to disseminate results with service providers, policymakers and communities so as to provide them with evidence of what women and their partners think about COVID-19, and suggestions for them to better provide services during future emergencies. Study results will be disseminated through online platforms, as well as publications on international peer-reviewed journals. This study aims to collaborate with potential research capacity strengthening for eligible partners in the country, including any National Research Council, national federation of professional specialties, etc. By participating in the data collection, analysis and interpretation, a group of young national scholars will be trained in qualitative research skills in the field of SRH.

Comment: For local adaptations, provide guidance on these risks by noting additional precautions and preparations required by study teams in countries where abortion is illegal and/or prohibited by religious law. This should include heightened sensitivity to protection of participants' confidentiality (women, men, and healthcare workers) and great care in de-identifying healthcare workers' comments regarding referral for termination in countries where illegal and/or subject to religious sanctions. The same applies for GBV.

For peer review only

Author´s contributions:
CRK and MA conceptualized and designed the protocol. JGC and LB performed the first round of adaptation of the protocol to this manuscript under the supervision of CRK and MA. All other authors participated with important suggestions for the proposed adaptation to local settings, read, and approved the final written manuscript.

Competing interests: None declared.

Funding statement:
This research has been supported by German Federal Ministry of Health (BMG) COVID-19 Research and development funding to WHO. It also has been supported by the UNDP/UNFPA/UNICEF/WHO/World Bank Special Programme of Research, Development and Research Training in Human Reproduction (HRP), a co-sponsored programme executed by the World Health Organisation (WHO).They are also funding the adaptation and implementation of the protocol in the HRP collaborating centres in Brazil, Burkina Faso, China, Ghana, Pakistan and Thailand. The views of the funding body have not influenced the content of this manuscript. This article represents the views of the named authors only and does not represent the views of their affiliated institutions.

References:

[1] Coronavirus disease (COVID-19) pandemic [Internet]. Who.int. 2020 [cited 25 May 2021]. Available from: https://www.who.int/emergencies/diseases/novel-coronavirus-2019

[2] China publishes timeline on COVID-19 information sharing, international cooperation [Internet, 2021]. [Cited 25 May 2021]. Available from: http://www.xinhuanet.com/english/2020-04/06/c_138951662.htm

[3] Cousins S. COVID-19 has "devastating" effect on women and girls. *Lancet* 2020; 396(1024):301-302.

[4] UNFPA. Technical Note. Impact of COVID-19 on Family Planning: What we know one year into the pandemic. [Internet, 11 March 2021]. [Cited 25 May 2021]. Available from: https://www.unfpa.org/sites/default/files/resource-pdf/COVID_Impact_FP_V5.pdf

[5] Chen H, Guo J, Wang C, *et al.* Clinical characteristics and intrauterine vertical transmission potential of COVID-19 infection in nine pregnant women: a retrospective review of medical records. The *Lancet*. 2020; 395(10226):809-815.

For peer review only - http://bmjopen.bmj.com/site/about/guidelines.xhtml

[6] Centeno-Tablante E, Medina-Rivera M, Finkelstein JL, *et al*. Transmission of SARS-CoV-2 through breast milk and breastfeeding: a living systematic review. *Ann N Y Acad Sci.* 2021; 1484(1):32-54.

[7] Edlow AG, Li JZ, Collier AY, *et al*. Assessment of maternal and neonatal SARS-CoV-2 viral load, transplacental antibody transfer, and placental pathology in pregnancies during the COVID-19 pandemic. *JAMA Netw Open* 2020; 3(2):e2030455.

[8] Gray KJ, Bordt EA, Atyeo C, *et al.* Coronavirus disease 2019 vaccine response in pregnant and lactaging women: a cohort study. *Am J Obstet Gynecol* 2021; XX:1e1-17. Doi: 10.1016/j.ajog.2021.03.023.

[9] Allotey J, Stallings E, Bonet M, *et al*. Clinical manifestations, risk factors, and maternal and perinatal outcomes of coronavirus disease 2019 in pregnancy: living systematic review and meta-analysis. *BMJ* 2020; 370:m3320.

[10] Millions more cases of violence, child marriage, female genital mutilation, unintended pregnancy expected due to the COVID-19 pandemic [Internet]. Unfpa.org. 2020 [cited 25 May 2021]. Available from: https://www.unfpa.org/news/millions-more-cases-violence-child-marriage-female-genital-mutilation-unintended-pregnancies#

[11] Barrow A. A Survey on Prevalence and Knowledge of Family Planning among Women of Childbearing Age in the Provincial Settings of the Gambia: A Descriptive Cross-Sectional Study. *Adv Prev Med* 2020; 2020: 8862290.

[12] Thobani R, Jessani S, Azam I, *et al*. Factors associated with the discontinuation of modern methods of contraception in the low income areas of Sukh Initiative Karachi: A community-based case control study. *PLoS ONE* 2019; 14(7): e0218952.

[13] Bateson DJ, Lohr PA, Norman WV, *et al*. The impact of COVID-19 on contraception and abortion care policy and practice: experiences from selected countries. *BMJ Sex Reprod Health*. 2020; 46(4):241-243.

[14] WHO. Service Availability and Readiness Assessment (SARA). Geneva: WHO, 2015. Available from: https://www.who.int/healthinfo/systems/sara_introduction/en/

[15] Fares-Otero NE, Pfaltz MC, Estrada-Lorenzo JM, *et al*. COVID-19: The need for screening for domestic violence and related neurocognitive problems. *J Psychiatr Res*. 2020; 130:433-434.

[16] Columbine M, Busza J, Cleland J, *et al*. Social science methods for research on sexual and reproductive health. Geneva, Switzerland: World Health Organization; 2012.

For peer review only - http://bmjopen.bmj.com/site/about/guidelines.xhtml

[17] Bronfenbrenne, U. *The ecology of human development*. Harvard University Press, 1979.

For peer review only

For peer review only - http://bmjopen.bmj.com/site/about/guidelines.xhtml

* **WHO HRP Social Science Research Team**: **Brazil** (Luis Bahamondes, Jose Guilherme Cecatti); **Burkina Faso** (Eunice Chomi, Seni Kouanda); **China** (Kun Tang, Yueping Guo, Yifang, Hanxiyue Zhang, Yifan Zhu, Ge Yang and Chunxiao Peng); **Ghana** (Deda Ogum Alangea, Kwasi Tropsey, Emefa Judith Modey); **Pakistan** (Rozina Karmaliani, Laila Ladak); **Thailand** (Pisake Lumbiganon, Jen Sothornwit); **WHO Secretariat** (Moazzam Ali, Caron Kim, Hamsadvani Kuganantham, Vanessa Brizuela, Anna Thorson, Joy Jerop Chebet, Hugo Gamerro Abrego, Soe Soe Thwin, Armando Seuc).

Acknowledgments
The authors thank the UNDP/UNFPA/UNICEF/WHO/World Bank Special Programme of Research, Development and Research Training in Human Reproduction (HRP), Department of Sexual and Reproductive Health and Research (SRH) and the World Health Emergency (WHE) programme from the World Health Organization (WHO) for leading and supporting this initiative of developing a generic protocol for such a qualitative study plus the publication of this manuscript. The authors also thank the overseas members of the hubs from the HRP Alliance who supported the initiative and gave important contributions to the protocol.

Disclaimer: The authors alone are responsible for the views expressed in this article and they do not necessarily represent the views, decisions or policies of the institutions with which they are affiliated.

Word count: 4162 words (max recommended 4000)

Figure legend:
Figure 1: The inductive logic of research in a qualitative study

For peer review only

Figure 1. The inductive logic of research in a qualitative study

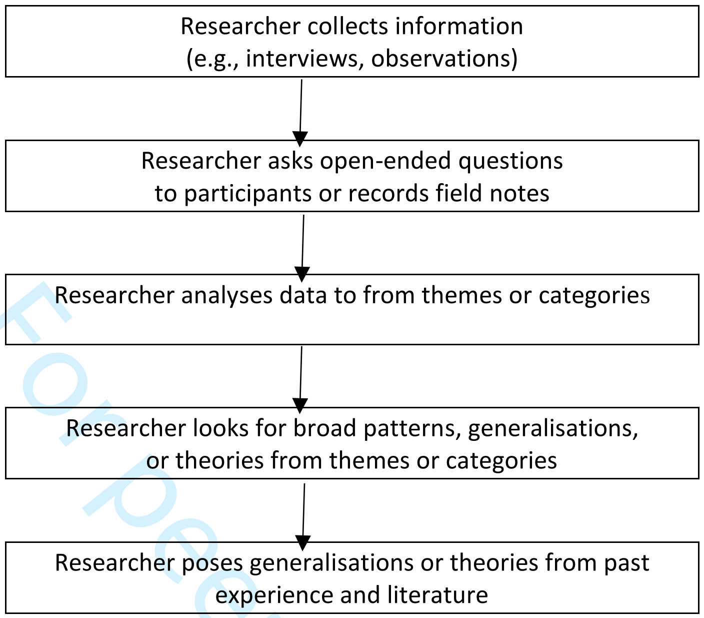

For peer review only

Legend of Supplementary files

Supplementary file S1: Study instrument--Interview Core Topic Guide

Supplementary file S2: Study instrument--Interview Detailed Topic Guide

Supplementary file S3: Written or oral informed consent form

Supplementary files:

Comment: Supplementary files 1 and 2 are only topic guides for helping interviewers to keep in mind the main content of the questions they should approach with each interviewee, and are not of course a questionnaire to be filled. Adaptations are possible and recommended for local and personal characteristics of the subject and language use can also be adapted for schooling level of the participant. Interview tools and the informed consent form would also need to be adjusted in settings where abortion is legally restrictive so that the questions are posed in a sensitive and non-judgemental manner. Given the inclusion of sensitive topics, it is essential that the study team implements all measures to protect the privacy and confidentiality of participants. The guidance document should provide potential options to countries on which questions to eliminate or modify to fit the local context. This should also be considered in the interview guide. Similar careful attention should be given to questions related to violence.

For peer review only

Supplementary file S1: Study instrument--Interview Core Topic Guide
Qualitative research on women's reproductive and psychosocial health in the context of COVID-19 - Core Topic Guide

1. Demographic information
    - Age
    - Ethnicity
    - Marital status
    - Socio-economic status
    - Occupation
    - Education status
    - Religion
    - Place of residence and housing condition
    - Obstetric characteristics (no. of pregnancies, parity)

2. Knowledge about COVID-19
    - Cause (sexual transmission)
    - Signs and symptoms of COVID-19
    - Care-seeking for COVID-19 infection
    - Sources of information and trusted communication channels (v. rumors, misinformation) Experience of COVID-19 (direct / indirect)
    - Perception of risk about being infected by COVID-19

3. Knowledge about pregnancy-related issues during COVID-19 (for pregnant participants)
    - Perception of risk associated with COVID-19 infection during pregnancy
    - Care-seeking for COVID-19 infection during pregnancy
    - Management of pregnancy with suspected COVID-19 infection

4. Prevention of COVID-19
    - Ways to prevent being infected by COVID-19 (probe at individual, household, community levels)
    - Responsibility to prevent COVID-19 (probe at individual, household, community levels, authorities)
    - Religious / socio-cultural practices and influencers
    - Perceptions of the vaccine

5. Sexual and reproductive health
    - Normal practices (including use of condoms)
    - Decision-making and agency to act
    - Issues of access / utilization including antenatal care
    - Challenges faced
6. Contraceptive practices
    - Changes in practice (if any) due to COVID-19
    - Who / what influenced the changes (if no changes in practices, why not)
    - Is pregnancy being avoided / delayed due to COVID-19?

For peer review only - http://bmjopen.bmj.com/site/about/guidelines.xhtml

- Access to/utilization of different methods of contraception (barriers / drivers)
- Challenges in access / utilization

7. Induced abortion
    - Perceptions about abortion at pre-COVID times
        - The role abortion plays in women's lives
        - Fear of stigma
        - Inequities in access to safe abortion
    - Perceptions about abortion during COVID-19
    - Influencing factors for perceptions and changes articulated
    - Perceptions of women if more women are seeking abortions due to COVID-19?
    - Methods of abortion (medical abortion or surgical abortion)
    - Access to different methods of abortion (barriers / drivers)
    - Challenges in accessing / utilizing safe abortion services (inequities in access)

8. Violence against women

For SRH healthcare providers

- Perceptions of changes in the nature and magnitude of violence against women, and especially domestic violence during COVID-19
- Nature of service provision during COVID-19, whether services for VAW are being changed or adapted and if safety considerations are adequately addressed?
- Access to /use of health services for VAW and related challenges

For pregnant and non-pregnant women

- Formal and informal systems of support, and coping strategies for VAW
- Perceptions of safety in home and in community safety/increased risk during COVID-19
- Women's expectations of services for gender-based violence
- What are additional stress factors (e.g. school-age children at home, older parents, loss of job/income, partner loss of job, etc.)
- How does stress affect frequency and severity of violence, particularly during COVID-19

9. Psycho-social
    - Psychosocial issues associated with being infected with COVID-19 (probe: more significant for women/men, more significant for pregnant women)

10. Final questions
    - On-going concerns about COVID-19 and its possible impact
    - Needs related to accurate information (probe further information needs – NB consider how to provide information if necessary)
    - Needs related to sexual reproductive health services (probe further SRH needs – NB consider how to support/provide if necessary)

Additional themes will be added as appropriate for each target group and questions revised accordingly, across the three phases of the study.

For peer review only - http://bmjopen.bmj.com/site/about/guidelines.xhtml

Supplementary file S2: Study instrument--Interview Detailed Topic Guide

Qualitative research on women's reproductive and psychosocial health in the context of COVID-19

Interview detailed topic guide for pregnant women

1. Demographic information

- Age
- Sex
- Ethnicity
- Marital status
- Annual income
- Occupation
- Education status
- Religion
- Place of residence and housing condition
- How many pregnancies did you have including current pregnancy?

2. Knowledge about COVID-19

- What did you know about the mode of transmission of the COVID-19 virus?
- What did you know about the signs and symptoms of COVID-19?
- Did you know where and how to seek care for COVID-19 infection? If yes, where and how?
- What's your sources of information about COVID-19? With whom do you communicate about COVID-19? How did you distinguish between facts and rumours/misinformation? Have you ever trusted rumours and misinformation during COVID-19? Please specify. How did you figure out what you trusted was wrong?
- Were you affected by COVID-19? Was any of your family members or friends/colleagues affected by COVID-19? How did being affected/having closed ones affected by COVID-19 affect your pregnancy? If you or your closed ones weren't affected by COVID-19, how did the epidemic/pandemic affect your pregnancy in general?
- What's your perception of risk about being infected by COVID-19? How did you estimate the risk?

3. Knowledge about pregnancy-related issues during COVID-19

- What's your perception of risk associated with COVID-19 infection during pregnancy? Are you aware of any risks associated with getting COVID-19 as a pregnant women? Please specify.
- Did you know where and how to seek care for COVID-19 infection during pregnancy? If yes, where and how? If you were ever affected by COVID-19, tell me your care-seeking experience.
- What did you know about the management of pregnancy with suspected COVID-19 infection? If you were ever infected by COVID-19, how did the infection challenge the management of your pregnancy by any means?

4. Prevention of COVID-19

For peer review only - http://bmjopen.bmj.com/site/about/guidelines.xhtml

- What did you know about the ways to prevent being infected by COVID-19? What about at individual levels? What about at household levels? What about at community levels? How did you practice these ways of prevention?
- Whom in your family was mainly responsible to practice prevention as a household? How do you think you, your household, your community, and the authorities fulfilled the responsibility to prevent COVID-19?
- How did you practice the prevention of COVID-19 differently than what is suggested by professionals due to religious/socio-cultural reasons (e.g. having gatherings due to religious reasons).
- What do you know about the COVID-19 vaccine?

5. Sexual and reproductive health

- Who (you, your partner, or your family member) usually make decision for various sexual and reproductive health related issues? Who is usually the agency to act?
- Did you have access to SRH services needed during COVID-19? If yes, how did you access to them? If not, what was the barrier? What did you do?
- What SRH-related challenges did you face during COVID-19? How did you cope with the challenges?

6. Contraceptive practices

- What is your normal practice of contraception?
- Was there any change in contraceptive practice among you and your partner due to COVID-19?
- If yes, what's the change? Who / what influenced the changes? If no, why not?
- Is your current pregnancy being delayed due to COVID-19?
- Did you have access to contraceptive methods needed during COVID-19? How many kinds of contraceptive methods did you have access to? Was there any barrier? What drives you to use this/these particular contraceptive method(s)?
- What other challenges did you meet in accessing / utilizing contraceptive methods? How did you cope with the challenges?

7. Induced abortion

- What's your perceptions about abortion during COVID-19? If you need to have abortion during COVID-19, will you opt for it? What challenges do you foresee in having abortion during COVID-19?
- What's your perceptions about abortion at typical times or rather, prior to COVID-19
- What were the influencing factors for your perceptions and changes articulated?

  - We have seen the increased need for reproductive health services such as abortion during this pandemic. Have you or someone you know ever had an abortion before? What role did it play in your life?
  - Were you fear of stigma when you have/intend to have/think of abortion?
  - Did you experience any inequities when you needed to access safe abortion?
- Did you seek abortion services during COVID-19? If yes, how many types of abortion methods were available? Was there any barrier of seeking safe abortion services during COVID-19? What's the driver for you to have the abortion and to have this particular kind of abortion?

For peer review only - http://bmjopen.bmj.com/site/about/guidelines.xhtml

For peer review only

- What other challenges did you meet in accessing / utilizing safe abortion services? How did you cope with the challenges?

8. Psycho-social
- Did you experience stress/anxiety/depression during COVID-19? How would rate your psychosocial health during your pregnancy? What role did COVID-19 play in influencing your psychosocial health during your pregnancy? Was it more significant for you or your partner?

9. Violence against women

For pregnant and non-pregnant women
- Formal and informal systems of support, and coping strategies for VAW
- Perceptions of safety in home and in community safety/increased risk during COVID-19
- Women's expectations of services for gender-based violence
- What are additional stress factors (e.g. school-age children at home, older parents, loss of job/income, partner loss of job, etc.)
- How does stress affect frequency and severity of violence, particularly during COVID-19

10. Final questions

- Do you have any concerns about COVID-19 currently? What's the impact on you?
- Was your need for accurate information fulfilled during COVID-19? What kind of accurate information you needed was missing during COVID-19? What do you think is the best way for you to access accurate information during COVID-19?
- Do you think you needs for sexual and reproductive health services were fulfilled during COVID-19? What kind of SRH services you needed wasn't met during COVID-19? What do you think is the best way the society can support the delivery of SRH services during emergencies like COVID-19?

Interview topic guide for non-pregnant women

1. Demographic information
- Age
- Sex
- Ethnicity
- Marital status
- Annual income
- Occupation
- Education status
- Religion
- Place of residence and housing condition

2. Knowledge about COVID-19
- What did you know about the mode of transmission of the COVID-19 virus?
- What did you know about the signs and symptoms of COVID-19?
- Did you know where and how to seek care for COVID-19 infection? If yes, where and how?

For peer review only - http://bmjopen.bmj.com/site/about/guidelines.xhtml

For peer review only

- What's your sources of information about COVID-19? With whom do you communicate about COVID-19? How did you distinguish between facts and rumours/misinformation? Have you ever trusted rumours and misinformation during COVID-19? How did you figure out what you trusted was wrong?
- Were you affected by COVID-19? Was any of your family members or friends/colleagues affected by COVID-19?
- What's your perception of risk about being infected by COVID-19? How did you estimate the risk?

3. Prevention of COVID-19

- What did you know about the ways to prevent being infected by COVID-19? What about at individual levels? What about at household levels? What about at community levels? How did you practice these ways of prevention?
- Whom in your family was mainly responsible to practice prevention as a household? Do you think you, your household, your community, and the authorities fulfilled the responsibility to prevent COVID-19?
- How did you practice the prevention of COVID-19 differently than what is suggested by professionals due to religious/socio-cultural reasons (e.g. having gatherings due to religious reasons).
- What are your thoughts/perceptions of the vaccine?

4. Sexual and reproductive health

- Who (you, your partner, or your family member) usually make decision for various sexual and reproductive health related issues? Who is usually the agency to act?
- Did you have access to SRH services needed during COVID-19? If yes, how did you access to them? If not, what was the barrier? What did you do?
- What SRH-related challenges did you face during COVID-19? How did you cope with the challenges?

5. Contraceptive practices

- What is your normal practice of contraception?
- Was there any change in contraceptive practice among you and your partner due to COVID-19?
- If yes, what's the change? Who / what influenced the changes? If no, why not?
- Did you have access to contraceptive methods needed during COVID-19? How many kinds of contraceptive methods did you have access to? Was there any barrier? What drives you to use this/these particular contraceptive method(s)?
- What other challenges did you meet in accessing / utilizing contraceptive methods? How did you cope with the challenges?

6. Induced abortion (applying the same changes as above)

- What's your perceptions about abortion during COVID-19? If you need to have abortion during COVID-19, will you opt for it? What challenges do you foresee in having abortion during COVID-19?
- What's your perceptions about abortion at typical times or rather, prior to COVID-19
- What were the influencing factors for your perceptions and changes articulated?

For peer review only

- ○ We have seen the increased need for reproductive health services such as abortion during this pandemic. Have you or someone you know ever had an abortion before? What role did it play in your life?
- ○ Were you fear of stigma when you have/intend to have/think of abortion?
- ○ Did you experience any inequities when you needed to access safe abortion?

- Did you seek abortion services during COVID-19? If yes, how many types of abortion methods were available? Was there any barrier of seeking safe abortion services during COVID-19? What's the driver for you to have the abortion and to have this particular kind of abortion?
- What other challenges did you meet in accessing / utilizing safe abortion services? How did you cope with the challenges?

7. Psycho-social

- Did you experience stress/anxiety/depression during COVID-19? How would rate your psychosocial health? What role did COVID-19 play in influencing your psychosocial health? Was it more significant for you or your partner?

8. Violence against women

For pregnant and non-pregnant women

- Formal and informal systems of support, and coping strategies for VAW
- Perceptions of safety in home and in community safety/increased risk during COVID-19
- Women's expectations of services for gender-based violence
- What are additional stress factors (e.g. school-age children at home, older parents, loss of job/income, partner loss of job, etc.)
- How does stress affect frequency and severity of violence, particularly during COVID-19

9. Final questions

- What concerns do you have about COVID-19 currently? What's the impact on you?
- Was your need for accurate information fulfilled during COVID-19? What kind of accurate information you needed was missing during COVID-19? What do you think is the best way for you to access accurate information during COVID-19?
- Do you think your needs for sexual and reproductive health services were fulfilled during COVID-19? What kind of SRH services you needed wasn't met during COVID-19? What do you think is the best way the society can support the delivery of SRH services during emergencies like COVID-19?

Interview topic guide for partners of pregnant and non-pregnant women

1. Demographic information

- Age
- Sex
- Ethnicity
- Marital status
- Annual income
- Occupation
- Education status

For peer review only - http://bmjopen.bmj.com/site/about/guidelines.xhtml

- Religion
- Place of residence and housing condition
- How many pregnancies did your partner have including current pregnancy?

2. Knowledge about COVID-19

- What did you know about the mode of transmission of the COVID-19 virus?
- What did you know about the signs and symptoms of COVID-19?
- Did you know where and how to seek care for COVID-19 infection? If yes, where and how?
- What's your sources of information about COVID-19? With whom do you communicate about COVID-19? How did you distinguish between facts and rumours/misinformation? Have you ever trusted rumours and misinformation during COVID-19? How did you figure out what you trusted was wrong?
- Were you affected by COVID-19? Was any of your family members or friends/colleagues affected by COVID-19? How did being affected/having closed ones affected by COVID-19 affect your partner's pregnancy? If you or your closed ones weren't affected by COVID-19, how did the epidemic/pandemic affect your partner's pregnancy in general?
- What's your perception of risk about being infected by COVID-19? How did you estimate the risk?

3. Knowledge about pregnancy-related issues during COVID-19 (For participants whose partners were pregnant during COVID-19)

- What's your perception of risk associated with COVID-19 infection during pregnancy? Are you aware of any risks associated with getting COVID-19 as a pregnant women?
- Did you know where and how to seek care for COVID-19 infection during pregnancy? If yes, where and how? If your partner was ever affected by COVID-19, tell me your care-seeking experience.
- What did you know about the management of pregnancy with suspected COVID-19 infection? If your partner was ever infected by COVID-19, did the infection challenge the management of her pregnancy by any means?

4. Prevention of COVID-19

- What did you know about the ways to prevent being infected by COVID-19? What about at individual levels? What about at household levels? What about at community levels? How did you practice these ways of prevention?
- Whom in your family was mainly responsible to practice prevention as a household? Do you think you, your household, your community, and the authorities fulfilled the responsibility to prevent COVID-19?
- How did you practice the prevention of COVID-19 differently than what is suggested by professionals due to religious/socio-cultural reasons (e.g. having gatherings due to religious reasons).

5. Sexual and reproductive health

- Who (you, your partner, or your family member) usually make decision for various sexual and reproductive health related issues? Who is usually the agency to act?
- Did your partner have access to SRH services needed during COVID-19? If yes, how did she access to them? If not, what was the barrier? What did you and your partner do?
- What SRH-related challenges did you and your partner face during COVID-19? How did you cope with the challenges?

For peer review only - http://bmjopen.bmj.com/site/about/guidelines.xhtml

6. Contraceptive practices
- What is your normal practice of contraception?
- Was there any change in contraceptive practice among you and your partner due to COVID-19?
- If yes, what's the change? Who / what influenced the changes? If no, why not?
- Is your partner's current pregnancy being delayed due to COVID-19?
- Did you have access to contraceptive methods needed during COVID-19? How many kinds of contraceptive methods did you have access to? Was there any barrier? What drives you to use this/these particular contraceptive method(s)?
- What other challenges did you and your partner meet in accessing / utilizing contraceptive methods? How did you cope with the challenges?

7. Induced abortion
- What's your perceptions about abortion at normal times?
- Have your partner ever had abortion before? What role did it play in your life?
- What's your perceptions about abortion during COVID-19? If your partner need to have abortion during COVID-19, will you opt for it? What challenges do you foresee in having abortion during COVID-19?
- What were the influencing factors for your perceptions and changes articulated?
- Did your partner seek abortion services during COVID-19? If yes, how many types of abortion methods were available? Was there any barrier of seeking safe abortion services during COVID-19? What's the driver for you and your partner to have the abortion and to have this particular kind of abortion?
- What other challenges did you and your partner meet in accessing / utilizing safe abortion services? How did you cope with the challenges?

8. Psycho-social
- How did you and your partner experience stress/anxiety/depression during COVID-19?
- If your partner is pregnant, how would you rate your and your partner's psychosocial health during her pregnancy? What role did COVID-19 play in influencing your and your partner's psychosocial health during her pregnancy? Was it more significant for you or your partner?
- If your partner is not pregnant, how would rate your and your partner's psychosocial health? What role did COVID-19 play in influencing your and your partner's psychosocial health? Was it more significant for you or your partner?

9. Final questions
- What concerns about COVID-19 do you have currently? What's the impact on you?
- Was your need for accurate information fulfilled during COVID-19? What kind of accurate information you needed was missing during COVID-19? What do you think is the best way for you to access accurate information during COVID-19?
- Do you think your and your partner's needs for sexual and reproductive health services were fulfilled during COVID-19? What kind of SRH services you needed wasn't met during COVID-19? What do you think is the best way the society can support the delivery of SRH services during emergencies like COVID-19?

For peer review only

Interview topic guide for health care professionals

1. Demographic information
- Age
- Sex
- Ethnicity
- Marital status
- Annual income
- Occupation
- Education status
- Religion
- Place of residence and housing condition

2. Knowledge about COVID-19
- What did you know about the mode of transmission of the COVID-19 virus?
- What did you know about the signs and symptoms of COVID-19?
- Did you know where and how to seek care for COVID-19 infection? If yes, where and how?
- What's your sources of information about COVID-19? With whom do you communicate about COVID-19? How did you distinguish between facts and rumours/misinformation? Have you ever trusted rumours and misinformation during COVID-19? How did you figure out what you trusted was wrong?
- Were you affected by COVID-19? Was any of your family members or friends/colleagues affected by COVID-19?
- What's your perception of risk about being infected by COVID-19? How did you estimate the risk?

3. Knowledge about pregnancy-related issues during COVID-19
- What's your perception of risk associated with COVID-19 infection during pregnancy?
- Where and how can people seek care for COVID-19 infection during pregnancy?
- What did you know about the management of pregnancy with suspected COVID-19 infection?

4. Prevention of COVID-19
- What did you know about the ways to prevent being infected by COVID-19? What about at individual levels? What about at household levels? What about at community levels?

5. Sexual and reproductive health
- Where and how can people seek SRH services needed during COVID-19? How was the utilization of SRH services in your hospital / clinic?
- What challenges did you meet in providing SRH services during COVID-19?

6. Contraceptive practices
- Where and how can people seek contraceptive methods during COVID-19? How many kinds of contraceptive methods were available in your hospital / clinic during COVID-19? How was the utilization of contraceptive services in your hospital / clinic?
- What challenges did you meet in providing contraceptive methods during COVID-19?

7. Induced abortion

For peer review only - http://bmjopen.bmj.com/site/about/guidelines.xhtml

- What's your perceptions about abortion at normal times?
- What's your perceptions about abortion during COVID-19?
- What were the influencing factors for your perceptions and changes articulated?
- Are more women seeking abortions due to COVID-19?
- How many methods of abortion are available in your hospital / clinic during COVID-19 and now? What kind of abortion do you suggest during COVID-19 and now? Why?
- What challenges did you meet in providing safe abortion services during COVID-19?

8. Psycho-social
- How did you experience stress/anxiety/depression during COVID-19? How would rate your psychosocial health? What role did COVID-19 play in influencing your psychosocial health?

9. Violence against women

For SRH healthcare providers
- Perceptions of changes in the nature and magnitude of violence against women, and especially domestic violence during COVID-19
- Nature of service provision during COVID-19, whether services for VAW are being changed or adapted and if safety considerations are adequately addressed?
- Access to /use of health services for VAW and related challenges

10. Final questions
- What concerns about COVID-19 do you have currently? What's the impact on you?
- What accurate information do you think should be provided to the society during COVID-19? What do you think is the best way to provide accurate information during emergencies like COVID-19?
- What sexual and reproductive health services do you think should be provided during COVID-19? What do you think is the best way the society can support the delivery of SRH services during emergencies like COVID-19?

For centres deciding on the inclusion of GBV in the main topics to be assessed in the study:

I'd like to hear your thoughts on how violence against women, and especially violence in the home, may be changing during this pandemic and under movement restrictions, and how the health system is responding to violence during the crisis.

A. Have you noticed any change in the number of women coming into the health centre reporting gender-based violence since the COVID-19 crisis began?
   - Follow up: Increase or decrease?
   - Follow up: Have the women coming in who report experiencing violence mostly been returning patients, or mostly been new patients?
   - Follow up: Have you noticed an increase in the frequency of abuse that women are experiencing since movement restrictions were put in place?
   - If possible, specify types of violence (physical, sexual, psychological by a partner), non-partner sexual abuse, other

B. Do you think that the violence women have been experiencing has become more severe

For peer review only - http://bmjopen.bmj.com/site/about/guidelines.xhtml

during the crisis, for example has there been an increase in injuries? Violence during pregnancy?

- Follow up: Have the types of violence that women are reporting changed? How so?

C. Since the start of COVID-19, has your health centre changed or adapted services for survivors ‘of violence?

- Probe: (if not mentioned) In some health centres, providers have maintained ‘case management’ using video calls, or text messages. Did your health centre make use of any new technologies to maintain services? If so did you develop a protocol to ensure safety of survivors?

D. Some health centres have had to deal with shortages of personal protective equipment and medications that have impacted their ability to offer services during the COVID-19 crisis. Have there been any new barriers to delivering services to survivors at your health centre?

E. In a lot of places, external referral systems have also been impacted by the crisis, such as lower numbers of available spaces in shelters due to the need for physical distancing, or NGOs that have been temporarily shut down. To your knowledge, have any changes have been made to your external referral systems? What are these?

- Follow up: Are any of the services to which you typically refer survivors available in a more limited capacity, or no longer available due to the crisis?

For peer review only

Supplementary file S3: Written or oral informed consent form
Informed consent (the original version will be presented in local language)

Model 1 (pregnant women)

Part One: Information Sheet

We would like to invite you to participate in the study "Qualitative Research on Pregnancy, Pregnancy Prevention and Induced Abortion in the Context of COVID-19". Doctor X is the investigator responsible for the study. The project focuses in analysing the psychosocial impacts of COVID-19 epidemic in reproductive decisions and health care for women, partners and health professionals. The researchers are particularly interested in comprehend the needs, attitudes and practices related to family planning / contraception, abortion, views and experience with violence, and health care during and after pregnancy in the context of the COVID-19 epidemic.

If you decide to participate in the study you will be asked to be interviewed in a series of two interviews - one will be carried out at recruitment and the second one at six months later. If you prefer for practical or security reasons to have the interview performed by telephone, this option will be followed. The interview is planned to be tape recorded. The interviewers will ask about the short- and long-term impacts of COVID-19 on your experience of pregnancy, pregnancy prevention, and induced abortion etc. The interview will be conducted at a convenient time and place for you. Each interview will last approximately 60-90 minutes. You will receive all the information needed before, during and after the completion of the research, and we assure you that your name will not be disclosed, or any information that can identify you or your family. The study and the corresponding data will not allow identification of the women interviewed. In this sense, during the process of writing down the content recorded during the interviews all identifying data will be omitted to guarantee that you cannot be identified. The interviews will also be numbered in a way that will not allow identification of individual participants. The recordings of the interviews will be put in a safe way and sent to an online location to be stored after the interview. Transcriptions will be kept under an online service for storing data, accessible only to the research team. All tape recordings will be kept in a secure place. We will remove the information that would allow your identification, and because of that, the record of the interviews will not be destroyed. These data will be kept for a period according to the local national rules. By giving your consent for this study, you are also allowing us to use the data you share with us to inform other ongoing and future initiatives in the field.

Your participation is voluntary. Eventually a small amount of money will be given to you to cover your expenses to attend the interview. You are free to refuse to participate, and you may withdraw your consent or discontinue participation at any time. If for any reason you discontinue your participation, your data collected until this moment can be used, otherwise, if you withdraw your consent, your data will be excluded and will not be used. The refusal to participate will not result in any penalty or loss of benefits. You can skip any questions you do not want to answer. All of the research data will be under the care of the principal investigator.

Possible risks: We are asking you to share with us some very personal and confidential information, and you may feel uncomfortable talking about some of the topics. You do not have to answer any question or take part in the discussion/interview/survey if you don't wish to do so, and that is also fine. You do not have to give us any reason for not responding to any question, or for refusing to take part in the interview.

Study benefits: At the end of the first round of the project, the research team will disseminate through online platform with information on family planning, sexual and reproductive health. The study will not directly benefit the participant, and any resources made available will also be available if they do not consent to participate.

This project has been reviewed and approved by the (*name of the local IRB*). If you have questions right now, we will be happy to clarify. During the research you can also contact (*name of one research assistant*) at telephone number *(cell phone number available for questions)* or at email address *(e-mail address available for questions)* for any questions.

For peer review only - http://bmjopen.bmj.com/site/about/guidelines.xhtml

Part Two: Certificate of Consent

I have read the foregoing information, or it has been read to me. I have had the opportunity to ask questions about it and any questions I have been asked have been answered to my satisfaction. I consent voluntarily to be a participant in this study

Print Name of Participant___________________
Signature of Participant ____________________
Date _____________________________
Day/month/year

If illiterate

I have witnessed the accurate reading of the consent form to the potential participant, and the individual has had the opportunity to ask questions. I confirm that the individual has given consent freely.

Print name of witness_____________ Thumb print of participant
Signature of witness______________
Date _________________________
Day/month/year

Statement by the researcher/person taking consent

I have accurately read out the information sheet to the potential participant, and to the best of my ability made sure that the participant understands that the following will be done:

1. Semi-structured interview

I confirm that the participant was given an opportunity to ask questions about the study, and all the questions asked by the participant have been answered correctly and to the best of my ability. I confirm that the individual has not been coerced into giving consent, and the consent has been given freely and voluntarily.

A copy of this ICF has been provided to the participant.
Print Name of Researcher/person taking the consent_________________________
Signature of Researcher /person taking the consent___________________________
Date _____________________________
Day/month/year

For peer review only

Model 2 (non-pregnant women, partners and health care professionals)

Part One: Information Sheet

We would like to invite you to participate in the study "Qualitative Research on Pregnancy, Pregnancy Prevention and Induced Abortion in the Context of COVID-19". Doctor X is the investigator responsible for the study. The project focuses in analysing the psychosocial impacts of COVID-19 epidemic in reproductive decisions and health care for women, partners and health professionals. The researchers are particularly interested in comprehend the needs, attitudes and practices related to family planning / contraception, abortion, views and experience with violence and health care in the context of the COVID-19 epidemic.

If you decide to participate in the study you will be asked to be interviewed. The interview will be conducted at a convenient time and place for you. The interview will be held in one meeting, and will last approximately 60-90 minutes. If you prefer for practical or security reasons to have the interview performed by telephone, this option will be followed. The interview is planned to be tape recorded. You will receive all the information needed before, during and after the completion of the research, and we assure you that your name will not be disclosed, or any information that can identify you or your family. The study and the corresponding data will not allow identification of the women interviewed. In this sense, during the process of writing down the content recorded during the interviews all identifying data will be omitted to guarantee that you cannot be identified.. The interviews will also be numbered in a way that will not allow identification of individual participants. The recordings of the interviews will be put in a safe way and sent to an online location to be stored after the interview. Transcriptions will be kept under an online service for storing data, accessible only to the research team. All tape recordings will be kept in a secure place. We will remove the information that would allow your identification, and because of that, the record of the interviews will not be destroyed. These data will be kept for a period according to the local national rules. By giving your consent for this study, you are also allowing us to use the data you share with us to inform other ongoing and future initiatives in the field.

Your participation is voluntary. Eventually a small amount of money will be given reimbursement you to cover your incidental expenses to attend the interview. You are free to refuse to participate, and you may withdraw your consent or discontinue participation at any time. If for any reason you discontinue your participation, your data collected until this moment can be used, otherwise, if you withdraw your consent, your data will be excluded and will not be used. The refusal to participate will not result in any penalty or loss of benefits. You can skip any questions you do not want to answer. All of the research data will be under the care of the principal investigator.

Possible risks: We are asking you to share with us some very personal and confidential information, and you may feel uncomfortable talking about some of the topics. You do not have to answer any question or take part in the discussion/interview/survey if you don't wish to do so, and that is also fine. You do not have to give us any reason for not responding to any question, or for refusing to take part in the interview.

Study benefits: At the end of the first round of the project the research team will disseminate through online platform with information on family planning, sexual and reproductive health. The study will not directly benefit the participant, and any resources made available will also be available if they do not consent to participate.

This project has been reviewed and approved by the (*name of the local IRB*). If you have questions right now, we will be happy to clarify. During the research you can also contact (*name of one research assistant*) at telephone number *(cell phone number available for questions)* or at email address *(e-mail address available for questions)* for any questions.

For peer review only - http://bmjopen.bmj.com/site/about/guidelines.xhtml

Part Two: Certificate of Consent

I have read the foregoing information, or it has been read to me. I have had the opportunity to ask questions about it and any questions I have been asked have been answered to my satisfaction. I consent voluntarily to be a participant in this study

Print Name of Participant__________________
Signature of Participant ___________________
Date ____________________________
Day/month/year

If illiterate

I have witnessed the accurate reading of the consent form to the potential participant, and the individual has had the opportunity to ask questions. I confirm that the individual has given consent freely.

Print name of witness____________ Thumb print of participant
Signature of witness_____________
Date _________________________
Day/month/year

Statement by the researcher/person taking consent

I have accurately read out the information sheet to the potential participant, and to the best of my ability made sure that the participant understands that the following will be done:

1. Semi-structured interview

I confirm that the participant was given an opportunity to ask questions about the study, and all the questions asked by the participant have been answered correctly and to the best of my ability. I confirm that the individual has not been coerced into giving consent, and the consent has been given freely and voluntarily.

A copy of this ICF has been provided to the participant.
Print Name of Researcher/person taking the consent________________________
Signature of Researcher /person taking the consent__________________________
Date ____________________________
Day/month/year

For peer review only

For peer review only - http://bmjopen.bmj.com/site/about/guidelines.xhtml

BMJ Open

Issues related to Pregnancy, Pregnancy Prevention and Abortion in the Context of the COVID-19 pandemic: a WHO qualitative study protocol

Journal:	*BMJ Open*
Manuscript ID	bmjopen-2022-063317.R1
Article Type:	Protocol
Date Submitted by the Author:	15-Sep-2022
Complete List of Authors:	Cecatti, Jose; University of Campinas, Obstetrics and Gynecology<br>Bahamondes, Luis; State University of Campinas – UNICAMP, Obst & Gynecol<br>Ali, Moazzam; Organisation mondiale de la Sante,<br>Alangea, Deda Ogum; University of Ghana College of Health Sciences, Population, Family & Reproductive Health<br>Brizuela, Vanessa; WHO, Department of Reproductive Health and Research<br>Nahyuha Chomi, Eunice; Institut de Recherche en Sciences de la Sante<br>Kouanda, Seni; Institut de Recherche en Sciences de la Sante<br>Karmaliani, Rozina; Aga Khan University School of Nursing & Midwifery, AKUSONAM<br>Ladak, Laila; Aga Khan University, School of Nursing and Midwifery<br>Lumbiganon, Pisake; Khon Kaen University, Faculty of Medicine<br>Emefa, Modey; University of Ghana<br>Jen, Sothornwit; Khon Kaen University, Department of Obstetrics and Gynaecology<br>Kuganantham, Hamsadvani; World Health Organization, Department of Reproductive Health and Research<br>Kim, Caron; World Health Organization, Sexual and Reproductive Health and Research<br>Collaborator Group, WHO HRP Social Science Research Team; World Health Organization
**Primary Subject Heading**:	Global health
Secondary Subject Heading:	Health services research, Public health, Qualitative research, Research methods, Obstetrics and gynaecology
Keywords:	Reproductive medicine < GYNAECOLOGY, COVID-19, QUALITATIVE RESEARCH

SCHOLARONE™
Manuscripts

RESEARCH PROTOCOL

Issues related to Pregnancy, Pregnancy Prevention and Abortion in the Context of the COVID-19 pandemic: a WHO qualitative study protocol

Jose Guilherme Cecatti [1]
Luis Bahamondes [1]
Moazzam Ali[2]
Deda Ogum Alangea[3]
Vanessa Brizuela[2]
Eunice Chomi[4]
Seni Kouanda[4]
Rozina Karmaliani[5]
Laila Ladak[5]
Pisake Lumbiganon[6]
Emefa J Modey[3]
Jen Sothornwit[6]
Hamsa Kuganantham[2]
Caron R Kim[2]

* On behalf of WHO HRP Social Science Research Team

1. Department of Obstetrics and Gynaecology from the University of Campinas and Cemicamp, Center for Research in Reproductive Health of Campinas, Brazil.
2. *UNDP-UNFPA-UNICEF-WHO-World Bank Special Programme of Research, Development and Research Training in Human Reproduction (HRP), a cosponsored programme executed by the World Health Organization (WHO)*, Geneva, Switzerland.
3. Dept. of Population, Family & Reproductive Health, School of Public Health, College of Health Sciences, University of Ghana, Akkra, Ghana.
4. Institut de Recherche en Sciences de la Sante, Ouagadougou, Burkina Faso.
5. Aga Khan University School of Nursing and Midwifery, Karachi, Pakistan.
6. Department of Ob & Gyn, Faculty of Medicine, Khon Kaen University, Khon Kaen, Thailand.

Corresponding author*:

Caron R Kim (kimca@who.int)
UNDP/UNFPA/UNICEF/WHO/World Bank Special Programme of Research, Development and Research Training in Human Reproduction (HRP)
Department of Sexual and Reproductive Health and Research
World Health Organization
Avenue Appia 20
Geneva, Switzerland

For peer review only - http://bmjopen.bmj.com/site/about/guidelines.xhtml

ABSTRACT

Introduction: The World Health Organisation (WHO) has generated standardised clinical and epidemiological research protocols to address key public health questions for SARS-CoV-2 (COVID-19) pandemic. We present a standardised protocol with the aim to fill a gap in understanding the needs, attitudes and practices related to sexual and reproductive health (SRH) in the context of COVID -19 pandemic, focusing on pregnancy, pregnancy prevention and abortion.

Methods and analysis plan: This protocol is a prospective qualitative research, using semi-structured interviews with at least 15 pregnant women at different gestational ages and after delivery, 6 months apart from the first interview. At least 10 male partners, 10 non-pregnant women and 5 healthcare professionals will be interviewed once during the course of the research. Higher number of subjects may be needed if a saturation is not achieved with these numbers. Data collection will be performed in a standardised way by skilled trained interviewers using written notes or audio-record of the interview. The data will be explored using the thematic content analysis and the researchers will look for broad patterns, generalisations, or theories from these categories.

Ethics and dissemination: The current protocol was first technically assessed and approved by the WHO scientific committee and then approved by its Ethics Review Committee as a guidance document. It is expected that each country/setting implementing such a generic protocol adapted to their conditions also obtain local ethical approval.

Comments for the user's consideration are provided in grey boxes throughout the document, as the user may need to modify methods slightly because of the local context in which this study will be carried out.

Key words: sexual and reproductive health; COVID-19 pandemic; psychosocial health; qualitative study

For peer review only

For peer review only - http://bmjopen.bmj.com/site/about/guidelines.xhtml

Strengths and limitations of this study:

- This is part of a series of WHO generic protocols with the purpose of facilitating and standardising the assessment of SRH issues during and after the COVID-19 pandemic in low and middle-income countries.
- With a generic protocol, adaptations of it are essential and encouraged, depending on the characteristics and resources from the countries/settings where they are planned to be implemented or other places deciding to do the same.
- One study limitation would be the difficulties and risks of different contexts of participating centres´ places regarding the COVID-19 pandemic at the moment of study implementation, which could introduce biases into subjects' perception.

For peer review only

INTRODUCTION

According to the World Health Organization (WHO), as of 26 September 2021, around 232 million cases and 4.7 million deaths of SARS-CoV-2 (Covid-19) cases have been confirmed globally. COVID-19 was declared as a public health emergency of international concern (PHEIC) at the end of January and characterized as a pandemic by WHO on 11st March 2020 [1].

China was the first country heavily affected by the pandemic since December 2019 [2], soon followed by several others in Asia, and then successively in Europe, America and Africa. To restrain the spread of COVID-19, domestic mobility was strictly regulated and in some affected areas transportation was restricted, with some differences in strategies adopted by different countries in different timeframes. In addition, physical distancing measures and isolation were firmly recommended in many countries and territories in different degrees, including closure of some basic services such as in-person classes at schools and universities, business activities, commerce, industry and even some healthcare services. As a result, there are some information indicating a disrupted healthcare service provision, including services in sexual and reproductive health (SRH) [3,4]. Despite the enormous medical and research resources directed towards the COVID-19 response, there are several gaps in the knowledge with regards to how the virus affects the body, even after recovery, and how the disease and the pandemic has affected people's lives.

Given SARS-CoV-2 novelty as virus of human transmission, there is limited evidence available to identify the impact of COVID -19 on SRH. At the start of the pandemic, there were questions whether or not the transmission of the virus or disease occurs from the pregnant woman to the fetus including at the early stage of fetal development [5]. The evidence has been growing on the risk of mother-to-child transmission, whether during pregnancy, childbirth or breastfeeding. [6-9]. In any case, the potential effects of COVID-19 infection during pregnancy on maternal and fetal health would influence women, their partners and healthcare professionals' decisions on reproductive healthcare.

According to the UNFPA, as health systems became overwhelmed with COVID-19 cases, clinical staff may not have had the time or personal protective equipment needed to provide contraceptive guidance and provision [10]. Women were and are refraining from visiting healthcare facilities due to movement restrictions or fears about COVID-19 exposure. Supply

For peer review only

For peer review only - http://bmjopen.bmj.com/site/about/guidelines.xhtml

chain disruptions are limiting the availability of contraceptives in many places. It is anticipated that significant levels of lockdown-related disruption over the months since the pandemic was declared could leave more than 47 million women in low- and middle-income countries unable to use modern contraceptives, leading to a projected 7 million additional unintended pregnancies [10].

In places in which medical termination of pregnancy is allowed, it has also been suggested that the demands for these services, including information provision have increased, which may be related to lack of contraceptive commodities or fear of unknown consequences of infection during pregnancy in the context of pandemic. There is some evidence from lower-resourced settings of women, especially those in an established relationship, having less control over the decision-making process of contraception or abortion [11,12]. Their husbands/partners and the extended family members have more control over their reproductive autonomy. It is important to examine how COVID-19 and the lack of resources during the pandemic influenced the decision-making process and the power dynamics involved in accessing contraception and abortion using information already available or implementing surveys for this [13,14].

Thus, it is important to assess the behavioural and psychosocial impacts of the COVID-19 pandemic on some aspects of women's SRH, especially among those who are pregnant, who are planning to become pregnant and those who want to avoid pregnancy, together with their partners and healthcare professionals, with a special attention to domestic and gender-based violence [15].

The current community-based protocol covers various SRH issues: abortion, contraception, pregnancy, sexual transmitted diseases (STD) prevention, domestic and gender-based violence (GBV), stigma, and discrimination. Covering multiple aspects of SRH will provide a better understanding of the *status quo* of SRH under the COVID-19 pandemic.

Comment: This section should be updated with country/setting-specific and updated data on COVID-19 epidemiology and current research findings prior to submission to local/national Institutional Review Boards (IRB) or donor agencies. Specific information regarding the health system management and provision of services should also be included in this section (facility-based childbirths, access to safe and legal abortion services, and access to contraception).

For peer review only - http://bmjopen.bmj.com/site/about/guidelines.xhtml

Problem statement, aims and objectives*:*

The global research question is on what is the impact of COVID-19 on the SRH of women[1] and their partners. The main aim is to increase knowledge and understanding of the behavioural and psychosocial impacts of the pandemic on women and men's SRH, especially those related to pregnancy, pregnancy prevention and abortion.

Their specific objectives are: 1) to investigate how the existence of the COVID-19 pandemic affects women's knowledge, attitudes, needs and practices on SRH services such as pregnancy care, pregnancy prevention, and abortion; 2) to explore how the pandemic affects men's and healthcare professionals' attitudes, needs and practices related to pregnancy, pregnancy prevention, and abortion; 3) to understand the knowledge and the perceptions of the study population on the COVID-19 impactand how this relates to their SRH-related attitudes and behaviours.

Comment: The SRH topics that the local study team intends to explore should be inserted in the objectives and may vary widely depending on the characteristics of the setting. Additional secondary objectives can be included, depending on the local context, in addition to the willingness of researchers and availability or resources to expand the current proposal.

METHODS

Study design

This study will use a phenomenological qualitative design with semi-structured interviews to collect data in either one point in time (for the subjects' partners and health workers) or two points in time (for women), 6-months apart. It is planned to be conducted in selected urban, semi-urban and possibly rural regions and will recruit a purposive sample of pregnant and non-pregnant subjects to enable researchers to explore women's knowledge, attitudes and practices in different temporal and situational contexts under COVID-19, including diversity in pregnancy status, SRH needs, and socioeconomic status [16]. In addition, their partners and local healthcare professionals will also be recruited to participate.

Study settings

[1] We recognize that cisgender women ,transgender men, nonbinary, gender-fluid and intersex individuals with a female reproductive system and capable of becoming pregnant may require SRH care. To facilitate readability of this protocol, when referring to all gender diverse people who may require SRH services, we used the word "women" most often, although we also use the term "subjects" and "individual".

For peer review only - http://bmjopen.bmj.com/site/about/guidelines.xhtml

Comment: there should be a brief description of the location – country, state, province, city – where the study is planned to be implemented, with some arguments justifying why this choice was made. Inform where and how pregnant women have access to antenatal care, where and how they deliver and access postnatal care, and if, where and how they get access to abortion and family planning services. State the number of health facilities chosen and their locations.

This protocol was developed to be implemented mainly in low and middle-income countries with institutions that have traditionally been partners in research with the WHO/HRP. However, any country or research centre can use the same protocol and they will be very welcomed. To capture variation in responses, health facilities in both rural and/or semi-urban/urban settings will be selected as focal points, including primary health unit or hospital where pregnant women are usually required to seek antenatal services. This enables the researchers' easy access to individuals attending the facility for interviews. The focal facilities will be selected purposively, using such criteria as available resources, COVID-19 pandemic status, availability of SRH services and the willingness of the healthcare providers to participate. The final selection of study sites will be confirmed jointly by the research team and local authorities, depending on the national procedures and regulations.

Comment: The implementation protocol should clearly define the catchment area of included health facilities and describe the demographics of the study sample. The strategy for getting help from health care providers at health facilities or other ways of identifying eligible subjects will rely on local characteristic of the health system and should be detailed described.

Study participants and sampling

The healthcare providers of the facilities selected as focal points will be asked to help the research team identify potential participants attending the facility. Pregnant individuals will be recruited at the local healthcare clinics when they access antenatal and/or SRH care services. Other participants, i.e., non-pregnant individual, partners of pregnant and non-pregnant individuals and healthcare professionals, will be recruited from these same selected facilities during clinical visits. To explore contraceptive choices, abortion care and pregnancy care, different women will need to be selected accordingly. Therefore, the location sampling method (venue based) will be used to recruit the participants [16], where participants can be

For peer review only - http://bmjopen.bmj.com/site/about/guidelines.xhtml

selected from places they attend, which can be identified in advance. This location sampling process is easily implemented in healthcare facilities where a sufficient number of available participants for the study are supposed to be present at an acceptable period of time.

Researchers will approach all potential participants following standard inclusion criteria. For pregnant women, the inclusion criteria include (1) age 18-45 years old; (2) living within the catchment area of the healthcare facility; (3) who were pregnant (ideally, in early pregnancy, in the first trimester) during the COVID-19 outbreak in that specific location (Level 3-4 on the WHO Situational Level Assessment); (4) either who have tested positive or negative for SARS-CoV-2 but currently not with an active infection (i.e., current PCR+ cases or within 2 weeks from the beginning of symptoms will not be recruited in the study while in this condition). For non-pregnant women, the inclusion criteria include: (1) aged 18-45 years old; (2) living within the catchment area of the facility; (3) who are not pregnant during and immediately after the COVID-19 outbreak in that location at the moment of enrolment; and (4) who are attending the health facility with the purpose of getting any service on SRH Women who do not meet the above criteria or who do not wish to take part will be excluded. Meanwhile, partners/family members of women in both groups attending the healthcare facility as companions and healthcare professionals from these facilities will be invited to participate in the interviews. Participation will be voluntary and only individuals who consent to participate after receiving information will be enrolled. Informed consent will be administered to the study participants in their local language highlighting the details about the study objectives and processes. Signed informed consents forms will be mandatory for face-to-face interviews. Otherwise, if interview is performed by phone or other electronic means, a verbal consent will be obtained; this will be necessary if the participant is not literate or has visual impairment. To account for the possible loss to follow-up of pregnant women, we will recruit 20% more participants than the intended sample size during the first interview.

Comment: The study will use gatekeepers who will be health service providers not participating in the study or community members who are known and trusted by the clients. Their main role will be to limited to identification and introduction of potential participants to the study team.

Sample size calculation

This study will recruit a sample of a minimum of 15 pregnant women per facility. Specifically, the suggested sample size is an approximation to the lower bound (the minimum number or participants required). However, the upper bound is determined by sampling until saturation,

For peer review only - http://bmjopen.bmj.com/site/about/guidelines.xhtml

which means they should be selected until it is found that similar answers are given to similar questions with no new themes emerging. The decision on saturation is expected to be taken according the experienced and trained interviewers/research assistants under the supervision of the local PI and other possible skilled participating researchers. Sample sizes can vary largely depending on the context that could interfere with the saturation of gathered information. In addition, at least 10 non-pregnant women, 10 partners (any gender, for both pregnant and non-pregnant women), and 5 healthcare professionals will also be recruited in each country. The minimum sample sizes were not determined by any specific criterion, but using a priori estimation. This was empirically decided as the minimum number of subjects in each category to provide meaningful information to be gathered with that from other countries/settings. A purposive sampling strategy aimed at saturation on the central study themes is appropriate for a qualitative study.

Comment: the sample size estimation should roughly follow the above-mentioned process, but adaptations are possible depending on the local characteristics and timeframe relative to the COVID-19 pandemic.

Adjustments should also be made if testing for infection is unavailable. Recruitment can be undertaken with assessment of infection status based on history (ie lack of symptoms or no report of being in close contact with someone with COVID-19)

Patient and public involvement

There was no patient or public involvement in the design of this protocol.

Data collection methods

This study includes a prospective series of two semi-structured interviews to be conducted with pregnant women and one semi-structured interview to be conducted with non-pregnant women, partners of both pregnant and non-pregnant women, and healthcare professionals in selected study areas. The reason for conducting two interviews with pregnant women is to explore the short-term and long-term impact and changes of antenatal care, abortion and potential experiences with childbirth. Each interview will last approximately 60 – 90 minutes. The script of the interviews will follow a topic guide and the researchers will promote a safe, convenient and comfortable environment to enable a comprehensive rapport. The topic-guide will be pre-tested to ensure question validity and assess interview length. During the interview, the research team will ask open-ended questions to participants and record field notes.

The process of participant recruitment aims to provide comprehensive information about the study as well as to build trust and rapport with the participants, as an essential step for

For peer review only - http://bmjopen.bmj.com/site/about/guidelines.xhtml

them to make a fully informed and voluntary decision. This process will be documented with a signed written or oral recorded informed consent. The contact information of the enrolled pregnant women will be asked (e.g., phone number or other forms of preferred contact), as well as that of other contacts (e.g., relatives) during the first interview to improve chances for follow-up for the second interview. Follow-up and the second interview will be performed in person or through phone/teleconference based on the participant's preference and need.

The interviews will ask about fertility desires, pregnancy, pregnancy prevention, abortion, and their related needs for accurate information and reproductive services. The first interview will capture how the above-mentioned factors, especially the needs for reproductive services were affected by COVID-19. The second interview will assess how the above-mentioned factors changed during the outbreak. The semi-structured interview allows for exploration of other SRH issues if these come up. The interviews will document pregnant women's knowledge, concerns, needs, attitudes and practices throughout the duration of their pregnancy on SRH, abortion, contraception, gender-based violence (GBV), etc.

A single semi-structured interview will be conducted with the other participants at one point in time. Interviews with non-pregnant women will document their knowledge, attitudes and practices towards SRH under the context of COVID-19. Interviews with partners will explore their knowledge, attitudes and concerns for their own and partner's SRH and wellbeing, including abortion. Depending on the context, local researchers may wish to interview extended family members (such as in-laws) in addition or instead of partners, as indicated by local customs. Interviews with healthcare professionals will focus on access to and availability of contraception and safe abortion; health-systems versus women's roles and responsibilities for prevention of pregnancy in the context of COVID-19; health system capacity to provide high-quality care for women; attitudes about pregnancy, contraception and abortion in the context of COVID-19; and perceived psychosocial effects on women, their families and local communities.

The study instruments were adapted from a core master guide built by WHO for qualitative research on women's reproductive and psychosocial health. In particular, questions were adapted to the context of COVID-19, but they should also include items relevant to the local settings. The instrument will be translated into the language of the location and pre-tested to ensure its validity and reliability with adjustments if needed. The interview core and detailed guides are present in Supplementary file S1 and S2.

For peer review only - http://bmjopen.bmj.com/site/about/guidelines.xhtml

Interviewers may have to confront difficult situations amongst infected participant cohort – like death or serious complications from COVID-19. The pandemic has changed the way the people live, exposed most of the population to unsafe and difficult situations which has affected many people personally (either because they themselves were sick or their close ones, or because they might have lost close ones to the disease). Similar sensitive situations could also appear regarding GBV and child abuse for instance. Ideally the study team members should have access to a mental healthcare provider for counselling and support to researchers and interviewees experiencing difficult situations. The country coordinating team should be able to provide the participants in the study with appropriate recommendations on how and where to access specific services if problems arise during the interviews.

Several changes occurred in the dynamics of the pandemic since its beginning. The protocol will probably be implemented in several different countries experiencing different stages of the pandemic. This is a potential factor to enrich the content of the findings from different settings. In addition, current experience on clinical and research activities show that such an interview is possible with personal protective equipment (PPE) or using interviewers who have already had COVID-19 or who were already vaccinated.

Comment: Depending on the local experience and availability of resources, the interviews should ideally be recorded and then appropriate data management with records, transcription, use of specific software and related topics should be adapted in the current protocol. If audio-recording is not an option, then data collection and recording of the interview will need to be adapted using hand notes. Additional themes can be added to the interview as appropriate for each target group and questions revised accordingly, across the whole study. Interview tools and the informed consent form would also need to be adjusted in settings where abortion is legally restrictive so that the questions are posed in a sensitive and non-judgemental manner. Given the inclusion of sensitive topics, it is essential that the study team implements all measures to protect the privacy and confidentiality of participants. The guidance document (supplementary files) should provide potential options to countries on which questions to eliminate or modify to fit the local context. This should also be considered in the interview guide. Similar careful attention should be given to questions related to violence.

Data Analyses

For peer review only - http://bmjopen.bmj.com/site/about/guidelines.xhtml

We adopt concepts from the Ecological Theory [17] to approach this research question. The Ecological Theory suggests that individual characteristics as well as the environment act interactively on one's personal development. In this context, individual characteristics refer to one's social-demographic characteristics, and one's knowledge and attitude related to SRH. The environment refers to a broad range of social and structural factors, including societal value, and access to SRH services. COVID-19 is the overarching environmental factor of the proposed study. This model serves as the framework for studying the knowledge, needs, attitudes and practices related to contraception, abortion, and healthcare before, during and after pregnancy in the context of the COVID-19 pandemic. This model will also guide study design, data collection, analysis and interpretation.

An inductive analytical technique will be used to analyse the data collected. Detailed examination of documents from interviews, either from written forms or transcription from recordings, will be conducted in order to generate initial categories and, above all, to suggest relationships between categories. Two researchers will do an initial screening of the interview transcripts and develop codes. During the process, the researchers will compile a list of themes and write short notes about important issues and patterns that are emerging. From these new themes, the researchers establish inductive codes. The researchers will then go through the transcripts again, and re-code them according to the new codes and themes. The researchers will also compile all the above-mentioned codes into a codebook. Two researchers will analyse the data concurrently either manually or with the help of a qualitative analysis software (such as NVivo®). The coding method and progress will be systematically discussed between the researchers and a third team member to ensure inter-coder reliability (ICR).

There are several optional techniques available for identifying core categories and their integration, such as data usage diagrams and written memos. Figure 1 provides a diagram with the elements of the procedures and process of applying the conceptual framework to this qualitative study.

There are different parties involved in this study as individuals living in COVID-19 pandemic areas during pregnancy, the SRH needs of non-pregnant women and their partners, including safe sex, contraception and abortion, as well as SRH healthcare providers. The participating partners in the study will be asked about their own SRH needs as well as those of their pregnant/not-pregnant partner.

For peer review only - http://bmjopen.bmj.com/site/about/guidelines.xhtml

Although the analysis is planned to provide a general full picture of SRH for women and men across all the participating countries/sites, the conceptual analysis will not be standardised across diverse contexts/countries due to their specificities and probable local adaptations for implementing the research protocol.

Data management and data access

Audio recordings and/or notes from the interviews will be transcribed verbatim, cross-checked as soon as feasible following the interview. Only the research team will have access to the data during the study. Single interviews will be de-identified at the time of transcription. Prospective interviews will be de-identified, transcribed, cross-checked, and summarised in order to inform subsequent interviews in the two-interview series. A participant ID will be assigned to each participant to ensure that data from the same participant is linked across two interview series. Data and transcriptions will be kept in encrypted hard drives, accessible only to the research team. All audio recordings will be kept in a secure place for the period of time as required for local country regulations and will then be destroyed. A subsample of the interview transcriptions will be translated into English (for those performed in a different language) to enable monitoring and inputs from WHO staff and consultants. All identifiable information, including telephone number and electronic accounts, which are used for contacting subjects during the interview, will be stored in a separate encrypted hard drive.

Ethics and dissemination

Respondents will provide a signed informed consent, also allowing eventually for a telephone interview (Supplementary file S3). The information collected will be de-identified. The identifiable information will be stored in a separate encrypted hard drive. The data will be kept in a safe storage way for the time as required by local national rules and laws on this topic. If additional analyses are planned, an appropriate permission should be obtained from the local ethics committees. If a participant decides to withdraw, the participantwill be discontinued and data already collected will be used. However, if the participant withdraws the consent, the respective data will be excluded and will not be used in the analysis.

Although the study is not planning to interview subjects currently with an active PCR+, all safety measures should be taken to minimise transmission risks. Masks will always be a requirement for both researchers and study participants and possibly other PPE and physical

For peer review only

distancing as locally required. In cases of suspected infection in either the researcher or the interviewee, they should be immediately referred to the health facility to be screened and tested, and the interview should be interrupted or postponed. If GBV is being assessed and women are identified in such a condition, social and legal advice should also be provided following the recommendations of local IRBs. In sites where abortions are illegal, discussions of access to abortion or post-abortion care may include risks to participants in terms of potential personal or legal repercussions.

There will be no financial incentives provided to participate in this study. However, the participants and their companions could receive compensation to cover the incidental costs of their participation, depending on the local regulations and availability of resources. This study protocol has received scientific approval through HRP's research project review panel (RP2) and ethics review committee (ERC) and, in accordance to local regulations, should be reviewed by a local ethics committee.

The results will be used to build an understanding of knowledge, practices, needs and attitudes related to fertility desires, pregnancy, pregnancy prevention and abortion under the context of COVID-19. They can help to improve health policies and SRH services. We aim to disseminate results with service providers, policymakers and communities so as to provide them with evidence of what women and their partners think about COVID-19, and suggestions for them to better provide services during future emergencies. Study results will be disseminated through online platforms, as well as publications on international peer-reviewed journals. This study aims to collaborate with potential research capacity strengthening for eligible partners in the country, including any National Research Council, national federation of professional specialties, etc. By participating in the data collection, analysis and interpretation, a group of young national scholars will be trained in qualitative research skills in the field of SRH.

Comment: For local adaptations, provide guidance on these risks by noting additional precautions and preparations required by study teams in countries where abortion is illegal and/or prohibited by religious law. This should include heightened sensitivity to protection of participants' confidentiality (women, men, and healthcare workers) and great care in de-identifying healthcare workers' comments regarding referral for termination in countries where illegal and/or subject to religious sanctions. The same applies for GBV.

For peer review only - http://bmjopen.bmj.com/site/about/guidelines.xhtml

Author´s contributions:

CRK and MA conceptualized and designed the protocol. JGC and LB performed the first round of adaptation of the protocol to this manuscript under the supervision of CRK and MA. All other authors, DA, VB, EC, SK, RK, LL, PL, EM, JS, HK, participated with important suggestions for the proposed adaptation to local settings, read, and approved the final written manuscript.

Competing interests: None declared.

Funding statement:

This research has been supported by the German Federal Ministry of Health (BMG Germany) COVID-19 Research and development funding to WHO (Award 70918). It also has been supported by the UNDP/UNFPA/UNICEF/WHO/World Bank Special Programme of Research, Development and Research Training in Human Reproduction (HRP), a co-sponsored programme executed by the World Health Organisation (WHO).They are also funding the adaptation and implementation of the protocol in the HRP collaborating centres in Brazil, Burkina Faso, China, Ghana, Pakistan and Thailand. The views of the funding body have not influenced the content of this manuscript. This article represents the views of the named authors only and does not represent the views of their affiliated institutions.

* **WHO HRP Social Science Research Team**: **Brazil** (Luis Bahamondes, Jose Guilherme Cecatti); **Burkina Faso** (Eunice Chomi, Seni Kouanda); **China** (Kun Tang, Yueping Guo, Yifang, Hanxiyue Zhang, Yifan Zhu, Ge Yang and Chunxiao Peng); **Ghana** (Deda Ogum Alangea, Kwasi Tropsey, Emefa Judith Modey); **Pakistan** (Rozina Karmaliani, Laila Ladak); **Thailand** (Pisake Lumbiganon, Jen Sothornwit); **WHO Secretariat** (Moazzam Ali, Caron Kim, Hamsadvani Kuganantham, Vanessa Brizuela, Anna Thorson, Joy Jerop Chebet, Hugo Gamerro Abrego, Soe Soe Thwin, Armando Seuc).

Acknowledgments

The authors thank the UNDP/UNFPA/UNICEF/WHO/World Bank Special Programme of Research, Development and Research Training in Human Reproduction (HRP), Department of Sexual and Reproductive Health and Research (SRH) and the World Health Emergency (WHE) programme from the World Health Organization (WHO) for leading and supporting this initiative of developing a generic protocol for such a qualitative study plus the publication of this manuscript. The authors also thank the overseas members of the hubs from the HRP Alliance who supported the initiative and gave important contributions to the protocol.

References:

[1] Coronavirus disease (COVID-19) pandemic [Internet]. Who.int. 2020 [cited 25 May 2021]. Available from: https://www.who.int/emergencies/diseases/novel-coronavirus-2019

For peer review only - http://bmjopen.bmj.com/site/about/guidelines.xhtml

[2] China publishes timeline on COVID-19 information sharing, international cooperation [Internet, 2021]. [Cited 25 May 2021]. Available from: http://www.xinhuanet.com/english/2020-04/06/c_138951662.htm

[3] Cousins S. COVID-19 has "devastating" effect on women and girls. *Lancet* 2020; 396(1024):301-302.

[4] UNFPA. Technical Note. Impact of COVID-19 on Family Planning: What we know one year into the pandemic. [Internet, 11 March 2021]. [Cited 25 May 2021]. Available from: https://www.unfpa.org/sites/default/files/resource-pdf/COVID_Impact_FP_V5.pdf

[5] Chen H, Guo J, Wang C, *et al.* Clinical characteristics and intrauterine vertical transmission potential of COVID-19 infection in nine pregnant women: a retrospective review of medical records. The *Lancet*. 2020; 395(10226):809-815.

[6] Centeno-Tablante E, Medina-Rivera M, Finkelstein JL, *et al*. Transmission of SARS-CoV-2 through breast milk and breastfeeding: a living systematic review. *Ann N Y Acad Sci.* 2021; 1484(1):32-54.

[7] Edlow AG, Li JZ, Collier AY, *et al*. Assessment of maternal and neonatal SARS-CoV-2 viral load, transplacental antibody transfer, and placental pathology in pregnancies during the COVID-19 pandemic. *JAMA Netw Open* 2020; 3(2):e2030455.

[8] Gray KJ, Bordt EA, Atyeo C, *et al.* Coronavirus disease 2019 vaccine response in pregnant and lactaging women: a cohort study. *Am J Obstet Gynecol* 2021; XX:1e1-17. Doi: 10.1016/j.ajog.2021.03.023.

[9] Allotey J, Stallings E, Bonet M, *et al*. Clinical manifestations, risk factors, and maternal and perinatal outcomes of coronavirus disease 2019 in pregnancy: living systematic review and meta-analysis. *BMJ* 2020; 370:m3320.

[10] Millions more cases of violence, child marriage, female genital mutilation, unintended pregnancy expected due to the COVID-19 pandemic [Internet]. Unfpa.org. 2020 [cited 25 May 2021]. Available from: https://www.unfpa.org/news/millions-more-cases-violence-child-marriage-female-genital-mutilation-unintended-pregnancies#

[11] Barrow A. A Survey on Prevalence and Knowledge of Family Planning among Women of Childbearing Age in the Provincial Settings of the Gambia: A Descriptive Cross-Sectional Study. *Adv Prev Med* 2020; 2020: 8862290.

For peer review only

[12] Thobani R, Jessani S, Azam I, *et al*. Factors associated with the discontinuation of modern methods of contraception in the low income areas of Sukh Initiative Karachi: A community-based case control study. *PLoS ONE* 2019; 14(7): e0218952.

[13] Bateson DJ, Lohr PA, Norman WV, *et al*. The impact of COVID-19 on contraception and abortion care policy and practice: experiences from selected countries. *BMJ Sex Reprod Health*. 2020; 46(4):241-243.

[14] WHO. Service Availability and Readiness Assessment (SARA). Geneva: WHO, 2015. Available from: https://www.who.int/healthinfo/systems/sara_introduction/en/

[15] Fares-Otero NE, Pfaltz MC, Estrada-Lorenzo JM, *et al*. COVID-19: The need for screening for domestic violence and related neurocognitive problems*. J Psychiatr Res*. 2020; 130:433-434.

[16] Columbine M, Busza J, Cleland J, *et al*. Social science methods for research on sexual and reproductive health. Geneva, Switzerland: World Health Organization; 2012.

[17] Bronfenbrenne, U. *The ecology of human development*. Harvard University Press, 1979.

For peer review only

For peer review only - http://bmjopen.bmj.com/site/about/guidelines.xhtml

Disclaimer: The authors alone are responsible for the views expressed in this article and they do not necessarily represent the views, decisions or policies of the institutions with which they are affiliated.

Word count: 4162 words (max recommended 4000)

Figure legend:
Figure 1: The inductive logic of research in a qualitative study

For peer review only

Figure 1. The inductive logic of research in a qualitative study

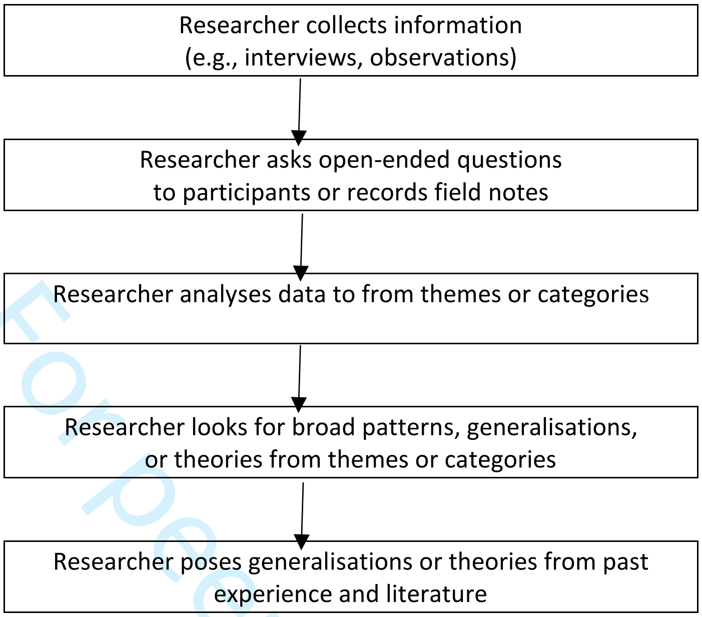

For peer review only

Legend of Supplementary files

Supplementary file S1: Study instrument--Interview Core Topic Guide

Supplementary file S2: Study instrument--Interview Detailed Topic Guide

Supplementary file S3: Written or oral informed consent form

Supplementary files:

Comment: Supplementary files 1 and 2 are only topic guides for helping interviewers to keep in mind the main content of the questions they should approach with each interviewee, and are not of course a questionnaire to be filled. Adaptations are possible and recommended for local and personal characteristics of the subject and language use can also be adapted for schooling level of the participant. Interview tools and the informed consent form would also need to be adjusted in settings where abortion is legally restrictive so that the questions are posed in a sensitive and non-judgemental manner. Given the inclusion of sensitive topics, it is essential that the study team implements all measures to protect the privacy and confidentiality of participants. The guidance document should provide potential options to countries on which questions to eliminate or modify to fit the local context. This should also be considered in the interview guide. Similar careful attention should be given to questions related to violence.

For peer review only

For peer review only - http://bmjopen.bmj.com/site/about/guidelines.xhtml

Supplementary file S1: Study instrument--Interview Core Topic Guide
Qualitative research on women's reproductive and psychosocial health in the context of COVID-19 - Core Topic Guide

1. Demographic information
   - Age
   - Ethnicity
   - Marital status
   - Socio-economic status
   - Occupation
   - Education status
   - Religion
   - Place of residence and housing condition
   - Obstetric characteristics (no. of pregnancies, parity)

2. Knowledge about COVID-19
   - Cause (sexual transmission)
   - Signs and symptoms of COVID-19
   - Care-seeking for COVID-19 infection
   - Sources of information and trusted communication channels (v. rumors, misinformation) Experience of COVID-19 (direct / indirect)
   - Perception of risk about being infected by COVID-19

3. Knowledge about pregnancy-related issues during COVID-19 (for pregnant participants)
   - Perception of risk associated with COVID-19 infection during pregnancy
   - Care-seeking for COVID-19 infection during pregnancy
   - Management of pregnancy with suspected COVID-19 infection

4. Prevention of COVID-19
   - Ways to prevent being infected by COVID-19 (probe at individual, household, community levels)
   - Responsibility to prevent COVID-19 (probe at individual, household, community levels, authorities)
   - Religious / socio-cultural practices and influencers
   - Perceptions of the vaccine

5. Sexual and reproductive health
   - Normal practices (including use of condoms)
   - Decision-making and agency to act
   - Issues of access / utilization including antenatal care
   - Challenges faced
6. Contraceptive practices
   - Changes in practice (if any) due to COVID-19
   - Who / what influenced the changes (if no changes in practices, why not)
   - Is pregnancy being avoided / delayed due to COVID-19?

For peer review only - http://bmjopen.bmj.com/site/about/guidelines.xhtml

- Access to/utilization of different methods of contraception (barriers / drivers)
- Challenges in access / utilization

7. Induced abortion
   - Perceptions about abortion at pre-COVID times
     - The role abortion plays in women's lives
     - Fear of stigma
     - Inequities in access to safe abortion
   - Perceptions about abortion during COVID-19
   - Influencing factors for perceptions and changes articulated
   - Perceptions of women if more women are seeking abortions due to COVID-19?
   - Methods of abortion (medical abortion or surgical abortion)
   - Access to different methods of abortion (barriers / drivers)
   - Challenges in accessing / utilizing safe abortion services (inequities in access)

8. Violence against women

For SRH healthcare providers

- Perceptions of changes in the nature and magnitude of violence against women, and especially domestic violence during COVID-19
- Nature of service provision during COVID-19, whether services for VAW are being changed or adapted and if safety considerations are adequately addressed?
- Access to /use of health services for VAW and related challenges

For pregnant and non-pregnant women

- Formal and informal systems of support, and coping strategies for VAW
- Perceptions of safety in home and in community safety/increased risk during COVID-19
- Women's expectations of services for gender-based violence
- What are additional stress factors (e.g. school-age children at home, older parents, loss of job/income, partner loss of job, etc.)
- How does stress affect frequency and severity of violence, particularly during COVID-19

9. Psycho-social
   - Psychosocial issues associated with being infected with COVID-19 (probe: more significant for women/men, more significant for pregnant women)

10. Final questions
    - On-going concerns about COVID-19 and its possible impact
    - Needs related to accurate information (probe further information needs – NB consider how to provide information if necessary)
    - Needs related to sexual reproductive health services (probe further SRH needs – NB consider how to support/provide if necessary)

Additional themes will be added as appropriate for each target group and questions revised accordingly, across the three phases of the study.

For peer review only

For peer review only - http://bmjopen.bmj.com/site/about/guidelines.xhtml

Supplementary file S2: Study instrument--Interview Detailed Topic Guide

Qualitative research on women's reproductive and psychosocial health in the context of COVID-19

Interview detailed topic guide for pregnant women

1. Demographic information
- Age
- Sex
- Ethnicity
- Marital status
- Annual income
- Occupation
- Education status
- Religion
- Place of residence and housing condition
- How many pregnancies did you have including current pregnancy?

2. Knowledge about COVID-19
- What did you know about the mode of transmission of the COVID-19 virus?
- What did you know about the signs and symptoms of COVID-19?
- Did you know where and how to seek care for COVID-19 infection? If yes, where and how?
- What's your sources of information about COVID-19? With whom do you communicate about COVID-19? How did you distinguish between facts and rumours/misinformation? Have you ever trusted rumours and misinformation during COVID-19? Please specify. How did you figure out what you trusted was wrong?
- Were you affected by COVID-19? Was any of your family members or friends/colleagues affected by COVID-19? How did being affected/having closed ones affected by COVID-19 affect your pregnancy? If you or your closed ones weren't affected by COVID-19, how did the epidemic/pandemic affect your pregnancy in general?
- What's your perception of risk about being infected by COVID-19? How did you estimate the risk?

3. Knowledge about pregnancy-related issues during COVID-19
- What's your perception of risk associated with COVID-19 infection during pregnancy? Are you aware of any risks associated with getting COVID-19 as a pregnant women? Please specify.
- Did you know where and how to seek care for COVID-19 infection during pregnancy? If yes, where and how? If you were ever affected by COVID-19, tell me your care-seeking experience.
- What did you know about the management of pregnancy with suspected COVID-19 infection? If you were ever infected by COVID-19, how did the infection challenge the management of your pregnancy by any means?

4. Prevention of COVID-19

For peer review only - http://bmjopen.bmj.com/site/about/guidelines.xhtml

- What did you know about the ways to prevent being infected by COVID-19? What about at individual levels? What about at household levels? What about at community levels? How did you practice these ways of prevention?
- Whom in your family was mainly responsible to practice prevention as a household? How do you think you, your household, your community, and the authorities fulfilled the responsibility to prevent COVID-19?
- How did you practice the prevention of COVID-19 differently than what is suggested by professionals due to religious/socio-cultural reasons (e.g. having gatherings due to religious reasons).
- What do you know about the COVID-19 vaccine?

5. Sexual and reproductive health

- Who (you, your partner, or your family member) usually make decision for various sexual and reproductive health related issues? Who is usually the agency to act?
- Did you have access to SRH services needed during COVID-19? If yes, how did you access to them? If not, what was the barrier? What did you do?
- What SRH-related challenges did you face during COVID-19? How did you cope with the challenges?

6. Contraceptive practices

- What is your normal practice of contraception?
- Was there any change in contraceptive practice among you and your partner due to COVID-19?
- If yes, what's the change? Who / what influenced the changes? If no, why not?
- Is your current pregnancy being delayed due to COVID-19?
- Did you have access to contraceptive methods needed during COVID-19? How many kinds of contraceptive methods did you have access to? Was there any barrier? What drives you to use this/these particular contraceptive method(s)?
- What other challenges did you meet in accessing / utilizing contraceptive methods? How did you cope with the challenges?

7. Induced abortion

- What's your perceptions about abortion during COVID-19? If you need to have abortion during COVID-19, will you opt for it? What challenges do you foresee in having abortion during COVID-19?
- What's your perceptions about abortion at typical times or rather, prior to COVID-19
- What were the influencing factors for your perceptions and changes articulated?

  - We have seen the increased need for reproductive health services such as abortion during this pandemic. Have you or someone you know ever had an abortion before? What role did it play in your life?
  - Were you fear of stigma when you have/intend to have/think of abortion?
  - Did you experience any inequities when you needed to access safe abortion?
- Did you seek abortion services during COVID-19? If yes, how many types of abortion methods were available? Was there any barrier of seeking safe abortion services during COVID-19? What's the driver for you to have the abortion and to have this particular kind of abortion?

For peer review only - http://bmjopen.bmj.com/site/about/guidelines.xhtml

- What other challenges did you meet in accessing / utilizing safe abortion services? How did you cope with the challenges?

8. Psycho-social
- Did you experience stress/anxiety/depression during COVID-19? How would rate your psychosocial health during your pregnancy? What role did COVID-19 play in influencing your psychosocial health during your pregnancy? Was it more significant for you or your partner?

9. Violence against women

For pregnant and non-pregnant women
- Formal and informal systems of support, and coping strategies for VAW
- Perceptions of safety in home and in community safety/increased risk during COVID-19
- Women's expectations of services for gender-based violence
- What are additional stress factors (e.g. school-age children at home, older parents, loss of job/income, partner loss of job, etc.)
- How does stress affect frequency and severity of violence, particularly during COVID-19

10. Final questions

- Do you have any concerns about COVID-19 currently? What's the impact on you?
- Was your need for accurate information fulfilled during COVID-19? What kind of accurate information you needed was missing during COVID-19? What do you think is the best way for you to access accurate information during COVID-19?
- Do you think you needs for sexual and reproductive health services were fulfilled during COVID-19? What kind of SRH services you needed wasn't met during COVID-19? What do you think is the best way the society can support the delivery of SRH services during emergencies like COVID-19?

Interview topic guide for non-pregnant women

1. Demographic information
- Age
- Sex
- Ethnicity
- Marital status
- Annual income
- Occupation
- Education status
- Religion
- Place of residence and housing condition

2. Knowledge about COVID-19
- What did you know about the mode of transmission of the COVID-19 virus?
- What did you know about the signs and symptoms of COVID-19?
- Did you know where and how to seek care for COVID-19 infection? If yes, where and how?

For peer review only - http://bmjopen.bmj.com/site/about/guidelines.xhtml
For peer review only

- What's your sources of information about COVID-19? With whom do you communicate about COVID-19? How did you distinguish between facts and rumours/misinformation? Have you ever trusted rumours and misinformation during COVID-19? How did you figure out what you trusted was wrong?
- Were you affected by COVID-19? Was any of your family members or friends/colleagues affected by COVID-19?
- What's your perception of risk about being infected by COVID-19? How did you estimate the risk?

3. Prevention of COVID-19

- What did you know about the ways to prevent being infected by COVID-19? What about at individual levels? What about at household levels? What about at community levels? How did you practice these ways of prevention?
- Whom in your family was mainly responsible to practice prevention as a household? Do you think you, your household, your community, and the authorities fulfilled the responsibility to prevent COVID-19?
- How did you practice the prevention of COVID-19 differently than what is suggested by professionals due to religious/socio-cultural reasons (e.g. having gatherings due to religious reasons).
- What are your thoughts/perceptions of the vaccine?

4. Sexual and reproductive health

- Who (you, your partner, or your family member) usually make decision for various sexual and reproductive health related issues? Who is usually the agency to act?
- Did you have access to SRH services needed during COVID-19? If yes, how did you access to them? If not, what was the barrier? What did you do?
- What SRH-related challenges did you face during COVID-19? How did you cope with the challenges?

5. Contraceptive practices

- What is your normal practice of contraception?
- Was there any change in contraceptive practice among you and your partner due to COVID-19?
- If yes, what's the change? Who / what influenced the changes? If no, why not?
- Did you have access to contraceptive methods needed during COVID-19? How many kinds of contraceptive methods did you have access to? Was there any barrier? What drives you to use this/these particular contraceptive method(s)?
- What other challenges did you meet in accessing / utilizing contraceptive methods? How did you cope with the challenges?

6. Induced abortion (applying the same changes as above)

- What's your perceptions about abortion during COVID-19? If you need to have abortion during COVID-19, will you opt for it? What challenges do you foresee in having abortion during COVID-19?
- What's your perceptions about abortion at typical times or rather, prior to COVID-19
- What were the influencing factors for your perceptions and changes articulated?

For peer review only - http://bmjopen.bmj.com/site/about/guidelines.xhtml

- We have seen the increased need for reproductive health services such as abortion during this pandemic. Have you or someone you know ever had an abortion before? What role did it play in your life?
- Were you fear of stigma when you have/intend to have/think of abortion?
- Did you experience any inequities when you needed to access safe abortion?

- Did you seek abortion services during COVID-19? If yes, how many types of abortion methods were available? Was there any barrier of seeking safe abortion services during COVID-19? What's the driver for you to have the abortion and to have this particular kind of abortion?
- What other challenges did you meet in accessing / utilizing safe abortion services? How did you cope with the challenges?

7. Psycho-social

- Did you experience stress/anxiety/depression during COVID-19? How would rate your psychosocial health? What role did COVID-19 play in influencing your psychosocial health? Was it more significant for you or your partner?

8. Violence against women

For pregnant and non-pregnant women

- Formal and informal systems of support, and coping strategies for VAW
- Perceptions of safety in home and in community safety/increased risk during COVID-19
- Women's expectations of services for gender-based violence
- What are additional stress factors (e.g. school-age children at home, older parents, loss of job/income, partner loss of job, etc.)
- How does stress affect frequency and severity of violence, particularly during COVID-19

9. Final questions

- What concerns do you have about COVID-19 currently? What's the impact on you?
- Was your need for accurate information fulfilled during COVID-19? What kind of accurate information you needed was missing during COVID-19? What do you think is the best way for you to access accurate information during COVID-19?
- Do you think your needs for sexual and reproductive health services were fulfilled during COVID-19? What kind of SRH services you needed wasn't met during COVID-19? What do you think is the best way the society can support the delivery of SRH services during emergencies like COVID-19?

Interview topic guide for partners of pregnant and non-pregnant women

1. Demographic information

- Age
- Sex
- Ethnicity
- Marital status
- Annual income
- Occupation
- Education status

For peer review only - http://bmjopen.bmj.com/site/about/guidelines.xhtml

- Religion
- Place of residence and housing condition
- How many pregnancies did your partner have including current pregnancy?

2. Knowledge about COVID-19

- What did you know about the mode of transmission of the COVID-19 virus?
- What did you know about the signs and symptoms of COVID-19?
- Did you know where and how to seek care for COVID-19 infection? If yes, where and how?
- What's your sources of information about COVID-19? With whom do you communicate about COVID-19? How did you distinguish between facts and rumours/misinformation? Have you ever trusted rumours and misinformation during COVID-19? How did you figure out what you trusted was wrong?
- Were you affected by COVID-19? Was any of your family members or friends/colleagues affected by COVID-19? How did being affected/having closed ones affected by COVID-19 affect your partner's pregnancy? If you or your closed ones weren't affected by COVID-19, how did the epidemic/pandemic affect your partner's pregnancy in general?
- What's your perception of risk about being infected by COVID-19? How did you estimate the risk?

3. Knowledge about pregnancy-related issues during COVID-19 (For participants whose partners were pregnant during COVID-19)

- What's your perception of risk associated with COVID-19 infection during pregnancy? Are you aware of any risks associated with getting COVID-19 as a pregnant women?
- Did you know where and how to seek care for COVID-19 infection during pregnancy? If yes, where and how? If your partner was ever affected by COVID-19, tell me your care-seeking experience.
- What did you know about the management of pregnancy with suspected COVID-19 infection? If your partner was ever infected by COVID-19, did the infection challenge the management of her pregnancy by any means?

4. Prevention of COVID-19

- What did you know about the ways to prevent being infected by COVID-19? What about at individual levels? What about at household levels? What about at community levels? How did you practice these ways of prevention?
- Whom in your family was mainly responsible to practice prevention as a household? Do you think you, your household, your community, and the authorities fulfilled the responsibility to prevent COVID-19?
- How did you practice the prevention of COVID-19 differently than what is suggested by professionals due to religious/socio-cultural reasons (e.g. having gatherings due to religious reasons).

5. Sexual and reproductive health

- Who (you, your partner, or your family member) usually make decision for various sexual and reproductive health related issues? Who is usually the agency to act?
- Did your partner have access to SRH services needed during COVID-19? If yes, how did she access to them? If not, what was the barrier? What did you and your partner do?
- What SRH-related challenges did you and your partner face during COVID-19? How did you cope with the challenges?

For peer review only - http://bmjopen.bmj.com/site/about/guidelines.xhtml

6. Contraceptive practices
- What is your normal practice of contraception?
- Was there any change in contraceptive practice among you and your partner due to COVID-19?
- If yes, what's the change? Who / what influenced the changes? If no, why not?
- Is your partner's current pregnancy being delayed due to COVID-19?
- Did you have access to contraceptive methods needed during COVID-19? How many kinds of contraceptive methods did you have access to? Was there any barrier? What drives you to use this/these particular contraceptive method(s)?
- What other challenges did you and your partner meet in accessing / utilizing contraceptive methods? How did you cope with the challenges?

7. Induced abortion
- What's your perceptions about abortion at normal times?
- Have your partner ever had abortion before? What role did it play in your life?
- What's your perceptions about abortion during COVID-19? If your partner need to have abortion during COVID-19, will you opt for it? What challenges do you foresee in having abortion during COVID-19?
- What were the influencing factors for your perceptions and changes articulated?
- Did your partner seek abortion services during COVID-19? If yes, how many types of abortion methods were available? Was there any barrier of seeking safe abortion services during COVID-19? What's the driver for you and your partner to have the abortion and to have this particular kind of abortion?
- What other challenges did you and your partner meet in accessing / utilizing safe abortion services? How did you cope with the challenges?

8. Psycho-social
- How did you and your partner experience stress/anxiety/depression during COVID-19?
- If your partner is pregnant, how would you rate your and your partner's psychosocial health during her pregnancy? What role did COVID-19 play in influencing your and your partner's psychosocial health during her pregnancy? Was it more significant for you or your partner?
- If your partner is not pregnant, how would rate your and your partner's psychosocial health? What role did COVID-19 play in influencing your and your partner's psychosocial health? Was it more significant for you or your partner?

9. Final questions
- What concerns about COVID-19 do you have currently? What's the impact on you?
- Was your need for accurate information fulfilled during COVID-19? What kind of accurate information you needed was missing during COVID-19? What do you think is the best way for you to access accurate information during COVID-19?
- Do you think your and your partner's needs for sexual and reproductive health services were fulfilled during COVID-19? What kind of SRH services you needed wasn't met during COVID-19? What do you think is the best way the society can support the delivery of SRH services during emergencies like COVID-19?

For peer review only

Interview topic guide for health care professionals

1. Demographic information
- Age
- Sex
- Ethnicity
- Marital status
- Annual income
- Occupation
- Education status
- Religion
- Place of residence and housing condition

2. Knowledge about COVID-19
- What did you know about the mode of transmission of the COVID-19 virus?
- What did you know about the signs and symptoms of COVID-19?
- Did you know where and how to seek care for COVID-19 infection? If yes, where and how?
- What's your sources of information about COVID-19? With whom do you communicate about COVID-19? How did you distinguish between facts and rumours/misinformation? Have you ever trusted rumours and misinformation during COVID-19? How did you figure out what you trusted was wrong?
- Were you affected by COVID-19? Was any of your family members or friends/colleagues affected by COVID-19?
- What's your perception of risk about being infected by COVID-19? How did you estimate the risk?

3. Knowledge about pregnancy-related issues during COVID-19
- What's your perception of risk associated with COVID-19 infection during pregnancy?
- Where and how can people seek care for COVID-19 infection during pregnancy?
- What did you know about the management of pregnancy with suspected COVID-19 infection?

4. Prevention of COVID-19
- What did you know about the ways to prevent being infected by COVID-19? What about at individual levels? What about at household levels? What about at community levels?

5. Sexual and reproductive health
- Where and how can people seek SRH services needed during COVID-19? How was the utilization of SRH services in your hospital / clinic?
- What challenges did you meet in providing SRH services during COVID-19?

6. Contraceptive practices
- Where and how can people seek contraceptive methods during COVID-19? How many kinds of contraceptive methods were available in your hospital / clinic during COVID-19? How was the utilization of contraceptive services in your hospital / clinic?
- What challenges did you meet in providing contraceptive methods during COVID-19?

7. Induced abortion

For peer review only - http://bmjopen.bmj.com/site/about/guidelines.xhtml

- What's your perceptions about abortion at normal times?
- What's your perceptions about abortion during COVID-19?
- What were the influencing factors for your perceptions and changes articulated?
- Are more women seeking abortions due to COVID-19?
- How many methods of abortion are available in your hospital / clinic during COVID-19 and now? What kind of abortion do you suggest during COVID-19 and now? Why?
- What challenges did you meet in providing safe abortion services during COVID-19?

8. Psycho-social

- How did you experience stress/anxiety/depression during COVID-19? How would rate your psychosocial health? What role did COVID-19 play in influencing your psychosocial health?

9. Violence against women

For SRH healthcare providers

- Perceptions of changes in the nature and magnitude of violence against women, and especially domestic violence during COVID-19
- Nature of service provision during COVID-19, whether services for VAW are being changed or adapted and if safety considerations are adequately addressed?
- Access to /use of health services for VAW and related challenges

10. Final questions

- What concerns about COVID-19 do you have currently? What's the impact on you?
- What accurate information do you think should be provided to the society during COVID-19? What do you think is the best way to provide accurate information during emergencies like COVID-19?
- What sexual and reproductive health services do you think should be provided during COVID-19? What do you think is the best way the society can support the delivery of SRH services during emergencies like COVID-19?

For centres deciding on the inclusion of GBV in the main topics to be assessed in the study:

I'd like to hear your thoughts on how violence against women, and especially violence in the home, may be changing during this pandemic and under movement restrictions, and how the health system is responding to violence during the crisis.

A. Have you noticed any change in the number of women coming into the health centre reporting gender-based violence since the COVID-19 crisis began?
    - Follow up: Increase or decrease?
    - Follow up: Have the women coming in who report experiencing violence mostly been returning patients, or mostly been new patients?
    - Follow up: Have you noticed an increase in the frequency of abuse that women are experiencing since movement restrictions were put in place?
    - If possible, specify types of violence (physical, sexual, psychological by a partner), non-partner sexual abuse, other

B. Do you think that the violence women have been experiencing has become more severe

For peer review only

during the crisis, for example has there been an increase in injuries? Violence during pregnancy?

- Follow up: Have the types of violence that women are reporting changed? How so?

C. Since the start of COVID-19, has your health centre changed or adapted services for survivors ‘of violence?

- Probe: (if not mentioned) In some health centres, providers have maintained ‘case management’ using video calls, or text messages. Did your health centre make use of any new technologies to maintain services? If so did you develop a protocol to ensure safety of survivors?

D. Some health centres have had to deal with shortages of personal protective equipment and medications that have impacted their ability to offer services during the COVID-19 crisis. Have there been any new barriers to delivering services to survivors at your health centre?

E. In a lot of places, external referral systems have also been impacted by the crisis, such as lower numbers of available spaces in shelters due to the need for physical distancing, or NGOs that have been temporarily shut down. To your knowledge, have any changes have been made to your external referral systems? What are these?

- Follow up: Are any of the services to which you typically refer survivors available in a more limited capacity, or no longer available due to the crisis?

For peer review only

For peer review only - http://bmjopen.bmj.com/site/about/guidelines.xhtml

Supplementary file S3: Written or oral informed consent form
Informed consent (the original version will be presented in local language)

Model 1 (pregnant women)
Part One: Information Sheet

We would like to invite you to participate in the study "Qualitative Research on Pregnancy, Pregnancy Prevention and Induced Abortion in the Context of COVID-19". Doctor X is the investigator responsible for the study. The project focuses in analysing the psychosocial impacts of COVID-19 epidemic in reproductive decisions and health care for women, partners and health professionals. The researchers are particularly interested in comprehend the needs, attitudes and practices related to family planning / contraception, abortion, views and experience with violence, and health care during and after pregnancy in the context of the COVID-19 epidemic.

If you decide to participate in the study you will be asked to be interviewed in a series of two interviews - one will be carried out at recruitment and the second one at six months later. If you prefer for practical or security reasons to have the interview performed by telephone, this option will be followed. The interview is planned to be tape recorded. The interviewers will ask about the short- and long-term impacts of COVID-19 on your experience of pregnancy, pregnancy prevention, and induced abortion etc. The interview will be conducted at a convenient time and place for you. Each interview will last approximately 60-90 minutes. You will receive all the information needed before, during and after the completion of the research, and we assure you that your name will not be disclosed, or any information that can identify you or your family. The study and the corresponding data will not allow identification of the women interviewed. In this sense, during the process of writing down the content recorded during the interviews all identifying data will be omitted to guarantee that you cannot be identified. The interviews will also be numbered in a way that will not allow identification of individual participants. The recordings of the interviews will be put in a safe way and sent to an online location to be stored after the interview. Transcriptions will be kept under an online service for storing data, accessible only to the research team. All tape recordings will be kept in a secure place. We will remove the information that would allow your identification, and because of that, the record of the interviews will not be destroyed. These data will be kept for a period according to the local national rules. By giving your consent for this study, you are also allowing us to use the data you share with us to inform other ongoing and future initiatives in the field.

Your participation is voluntary. Eventually a small amount of money will be given to you to cover your expenses to attend the interview. You are free to refuse to participate, and you may withdraw your consent or discontinue participation at any time. If for any reason you discontinue your participation, your data collected until this moment can be used, otherwise, if you withdraw your consent, your data will be excluded and will not be used. The refusal to participate will not result in any penalty or loss of benefits. You can skip any questions you do not want to answer. All of the research data will be under the care of the principal investigator.

Possible risks: We are asking you to share with us some very personal and confidential information, and you may feel uncomfortable talking about some of the topics. You do not have to answer any question or take part in the discussion/interview/survey if you don't wish to do so, and that is also fine. You do not have to give us any reason for not responding to any question, or for refusing to take part in the interview.

Study benefits: At the end of the first round of the project, the research team will disseminate through online platform with information on family planning, sexual and reproductive health. The study will not directly benefit the participant, and any resources made available will also be available if they do not consent to participate.

This project has been reviewed and approved by the (*name of the local IRB*). If you have questions right now, we will be happy to clarify. During the research you can also contact (*name of one research assistant*) at telephone number *(cell phone number available for questions)* or at email address *(e-mail address available for questions)* for any questions.

For peer review only - http://bmjopen.bmj.com/site/about/guidelines.xhtml

Part Two: Certificate of Consent

I have read the foregoing information, or it has been read to me. I have had the opportunity to ask questions about it and any questions I have been asked have been answered to my satisfaction. I consent voluntarily to be a participant in this study

Print Name of Participant__________________
Signature of Participant ___________________
Date ____________________________
Day/month/year

If illiterate

I have witnessed the accurate reading of the consent form to the potential participant, and the individual has had the opportunity to ask questions. I confirm that the individual has given consent freely.

Print name of witness____________ Thumb print of participant
Signature of witness_____________
Date _________________________
Day/month/year

Statement by the researcher/person taking consent

I have accurately read out the information sheet to the potential participant, and to the best of my ability made sure that the participant understands that the following will be done:

1. Semi-structured interview

I confirm that the participant was given an opportunity to ask questions about the study, and all the questions asked by the participant have been answered correctly and to the best of my ability. I confirm that the individual has not been coerced into giving consent, and the consent has been given freely and voluntarily.

A copy of this ICF has been provided to the participant.
Print Name of Researcher/person taking the consent________________________
Signature of Researcher /person taking the consent__________________________
Date ____________________________
Day/month/year

For peer review only

Model 2 (non-pregnant women, partners and health care professionals)

Part One: Information Sheet

We would like to invite you to participate in the study "Qualitative Research on Pregnancy, Pregnancy Prevention and Induced Abortion in the Context of COVID-19". Doctor X is the investigator responsible for the study. The project focuses in analysing the psychosocial impacts of COVID-19 epidemic in reproductive decisions and health care for women, partners and health professionals. The researchers are particularly interested in comprehend the needs, attitudes and practices related to family planning / contraception, abortion, views and experience with violence and health care in the context of the COVID-19 epidemic.

If you decide to participate in the study you will be asked to be interviewed. The interview will be conducted at a convenient time and place for you. The interview will be held in one meeting, and will last approximately 60-90 minutes. If you prefer for practical or security reasons to have the interview performed by telephone, this option will be followed. The interview is planned to be tape recorded. You will receive all the information needed before, during and after the completion of the research, and we assure you that your name will not be disclosed, or any information that can identify you or your family. The study and the corresponding data will not allow identification of the women interviewed. In this sense, during the process of writing down the content recorded during the interviews all identifying data will be omitted to guarantee that you cannot be identified.. The interviews will also be numbered in a way that will not allow identification of individual participants. The recordings of the interviews will be put in a safe way and sent to an online location to be stored after the interview. Transcriptions will be kept under an online service for storing data, accessible only to the research team. All tape recordings will be kept in a secure place. We will remove the information that would allow your identification, and because of that, the record of the interviews will not be destroyed. These data will be kept for a period according to the local national rules. By giving your consent for this study, you are also allowing us to use the data you share with us to inform other ongoing and future initiatives in the field.

Your participation is voluntary. Eventually a small amount of money will be given reimbursement you to cover your incidental expenses to attend the interview. You are free to refuse to participate, and you may withdraw your consent or discontinue participation at any time. If for any reason you discontinue your participation, your data collected until this moment can be used, otherwise, if you withdraw your consent, your data will be excluded and will not be used. The refusal to participate will not result in any penalty or loss of benefits. You can skip any questions you do not want to answer. All of the research data will be under the care of the principal investigator.

Possible risks: We are asking you to share with us some very personal and confidential information, and you may feel uncomfortable talking about some of the topics. You do not have to answer any question or take part in the discussion/interview/survey if you don't wish to do so, and that is also fine. You do not have to give us any reason for not responding to any question, or for refusing to take part in the interview.

Study benefits: At the end of the first round of the project the research team will disseminate through online platform with information on family planning, sexual and reproductive health. The study will not directly benefit the participant, and any resources made available will also be available if they do not consent to participate.

This project has been reviewed and approved by the (*name of the local IRB*). If you have questions right now, we will be happy to clarify. During the research you can also contact (*name of one research assistant*) at telephone number *(cell phone number available for questions)* or at email address *(e-mail address available for questions)* for any questions.

For peer review only - http://bmjopen.bmj.com/site/about/guidelines.xhtml

Part Two: Certificate of Consent

I have read the foregoing information, or it has been read to me. I have had the opportunity to ask questions about it and any questions I have been asked have been answered to my satisfaction. I consent voluntarily to be a participant in this study

Print Name of Participant__________________

Signature of Participant ___________________

Date ____________________________

Day/month/year

If illiterate

I have witnessed the accurate reading of the consent form to the potential participant, and the individual has had the opportunity to ask questions. I confirm that the individual has given consent freely.

Print name of witness____________ Thumb print of participant

Signature of witness_____________

Date _________________________

Day/month/year

Statement by the researcher/person taking consent

I have accurately read out the information sheet to the potential participant, and to the best of my ability made sure that the participant understands that the following will be done:

1. Semi-structured interview

I confirm that the participant was given an opportunity to ask questions about the study, and all the questions asked by the participant have been answered correctly and to the best of my ability. I confirm that the individual has not been coerced into giving consent, and the consent has been given freely and voluntarily.

A copy of this ICF has been provided to the participant.

Print Name of Researcher/person taking the consent________________________

Signature of Researcher /person taking the consent__________________________

Date ____________________________

Day/month/year

For peer review only - http://bmjopen.bmj.com/site/about/guidelines.xhtml
